# *In vivo* human embryonic spinal cord atlas validates stem cell–derived human dorsal interneurons and reveals ASD spinal signatures

**DOI:** 10.64898/2025.12.22.696129

**Published:** 2025-12-24

**Authors:** Sandeep Gupta, Eric Heinrichs, Cristian Rodriguez, Emily Friedman, Salena Gallardo, Talin Dermirjie, Teny Panosian, Keith Phan, Anooshik Tahmasian, Yahir Verdin, Samantha J. Butler

**Affiliations:** 1 Department of Neurobiology, David Geffen School of Medicine, University of California, Los Angeles Los Angeles, CA 90095; 2 Department of Cell Biology, University of Alberta, Edmonton, Canada; 3 Molecular Biology Interdepartmental Graduate Program, University of California, Los Angeles Los Angeles, CA 90095; 4 CIRM Bridges to Research Program, California State University, Northridge, CA 91330; 5 Eli and Edythe Broad Center of Regenerative Medicine and Stem Cell Research, University of California, Los Angeles Los Angeles, CA 90095; 6 Intellectual & Developmental Disabilities Research Center, University of California, Los Angeles Los Angeles, CA 90095

**Keywords:** human, stem cells, spinal cord, sensory, dorsal interneurons, reference atlas, directed differentiation protocol, neuromesodermal progenitors, BMP4, GDF11, HOX genes, posteriorization

## Abstract

Spinal cord injuries (SCI) result in the loss of motor and sensory function. We are working towards restoring sensation by developing directed differentiation protocols to generate dorsal spinal interneurons (dIs; dI1–dI6) from human embryonic stem cells (hESCs). Here, we present an improved method that produces human dIs via a neuromesodermal progenitor state, the physiological intermediate for spinal cord development. We show that retinoic acid (RA), bone morphogenetic protein 4 (BMP4) and growth differentiation factor (GDF) 11 direct dI identity, while GDF11 and extended time in culture promotes posterior spinal identities. Together, these protocols generate the full complement of dorsal subtypes along the entire anterior–posterior axis of the spinal cord. To benchmark *in vitro*-derived dIs, we constructed a single-cell RNA-Seq atlas of the human embryonic spinal cord and used it to show that hESC-derived dIs closely match their endogenous counterparts. The atlas also reveals that the dI4/dI5 populations dramatically expand in comparison with the other spinal lineages. Moreover, they have mechanosensory circuit signatures linked to autism spectrum disorder, implicating spinal circuits in autistic phenotypes.

## Introduction

Spinal cord injury (SCI) is a debilitating condition, with no cure^1^, making this condition one of the most pressing, unmet needs in modern medicine. Estimates of affected individuals range from 7 to 152 million individuals worldwide^2^. SCIs can occur at any stage of life, and are caused by a traumatic event^3, 4^ that results in damage to the spinal cord that then leads to disrupted connections with the brain^5–7^. While loss of motor function is the most well-known consequence of SCI, somatosensory perception is also impaired^8, 9^, such that patients lose the ability to sense pain, touch and proprioception. Spontaneous recovery is often limited; ~90% of individuals with complete paraplegia between T2 and L1 exhibit only minimal changes in sensory level within the first year, while sensory recovery in tetraplegia is highly variable^10^. Loss of somatosensation can be emotionally devasting for patients; however, the recovery of somatosensation remains understudied compared with motor control, despite its crucial role in everyday tasks and as a feedback regulator to the motor system.

Cellular replacement therapies have emerged as a promising regenerative approach to replace the tissue lost after SCI^11, 12^. Recent advances in stem-cell technologies permit the generation of specific neuronal subtypes to repair damaged circuits and re-establish functional connectivity^13–15^. However, these approaches have not yet been successfully implemented for SCI, perhaps because no therapy has leveraged the developmental logic of the spinal cord, to derive directed differentiation protocols, that accurate reflect the regional identity of the spinal neurons, particularly along the anterior–posterior axis. The spinal cord serves as a central relay center, conveying signals between the periphery and the brain^16^. Sensory signals enter the spinal cord through the dorsal root, while motor commands exit via the ventral root^16, 17^, thereby collectively forming 31 spinal nerves^18^. Each spinal nerve bundle connects to a specific segmental region along the anterior-posterior axis, innervating different muscles or visceral organs^19, 20^. Any regenerative therapy must re-establish these region-specific neuronal identities, since the location of the injury will determine the affected organs^21^. In particular, the restoration of bladder and bowel function is a key therapeutic goal, given its central importance for patient autonomy and quality of life.

The signals that determine regional identity have been well characterized in the developing spinal cord^22, 23^. Anterior/posterior identity is specified by the expression of *HOX* genes^23–25^. The *HOX* genes encode highly conserved homeodomain transcription factors that are organized into four chromosomal clusters, *HOXA*, *HOXB*, *HOXC,* and *HOXD*. *HOX* transcription is activated in a temporally-restricted manner^26–28^, with 3’ *HOX* genes expressed first to specify anterior fates^29–31^ while the 5’ HOX genes are progressively expressed later to define posterior identities^28^. Regional signaling centers, such as the roof plate, floor plate, and somites, secrete growth factors that pattern the early neural tube along the dorsal ventral axis^32^. In the dorsal spinal cord, six dorsal progenitor (dP) populations generate six classes of dorsal interneurons (dIs, dI1-dI6) that relay touch, pain and temperature perception, and proprioception^17^. In the ventral spinal cord, five classes of interneurons (v0, v1, v2a/b, v3) and motor neurons (MN) control locomotor output^33, 34^.

Many directed differentiation protocols exist to generate stem cell-derived spinal MNs, which have shown some promise recovering motor function in SCI models^35–37^. However, a complete recovery from SCI will also require protocols that derive dIs, from along the anterior-posterior axis. Early protocols for generating stem cell-derived spinal neurons recapitulated developmental patterning by first inducing anterior neuroepithelium (NE), followed by posteriorization towards spinal cord fates^38–41^. While these approaches generate MNs and a subset of dI populations with brachial-thoracic identities they fail to derive the most posterior spinal identities^38, 40^. Moreover, this is not the mechanism by which the posterior mammalian spinal cord arises *in vitro.* Rather, much of the spinal cord is derived from bipotential neuromesodermal progenitors (NMPs)^42^, which give rise to the lumbar-sacral circuits essential for bladder and bowel control^42, 43^. Consistent with this, NMP-based protocols show improved outcomes; a recent mouse embryonic stem cell (ESC) protocol that derived dIs through an NMP intermediate produced the full complement of dIs that transcriptionally and functionally resemble their endogenous counterparts^44, 45^. However, an equivalent human dI protocol has yet to be developed, and it remains unresolved whether dIs with the most posterior spinal identities can be generated.

A further challenge in the development of SCI therapies lies in accurately benchmarking human stem cell-derived spinal neurons. Directed differentiation protocols generate heterogenous neuronal repertoires, while the lack of a comprehensive human developmental reference atlas limits the direct validation of cellular identities^40^. Recent advances in single-cell (sc) sequencing technologies have led to a rapid expansion in the number and scale of publicly available datasets, enabling high-resolution comparisons between *in vitro*–derived cell types and their endogenous counterparts^46, 47^. In parallel, increasingly sophisticated computational methods now permit the integration of these disparate datasets into unified atlases to probe development and disease^48, 49^. Despite being applied across multiple fields, these approaches have only been applied sparingly to human spinal cord development. The existing datasets for the human spinal cord thus offer an incomplete view of neuronal diversity, developmental trajectories, and regional identities. The development of a comprehensive human spinal cord atlas would therefore provide a critical benchmark for validating stem cell differentiation protocols, enable identification of novel genes and cell types involved in somatosensory circuitry, and guide the rational design of region-specific therapeutic strategies for SCI. Collectively, these approaches offer a unique opportunity to directly investigate the human spinal cord in both during development and in disease states.

Here, we integrated six publicly available sc datasets^28, 50–54^ to generate a comprehensive transcriptomic reference atlas of the developing human spinal cord, spanning gestation weeks 4–25. This atlas reveals a marked expansion in the dI4/dI5 populations, which encode pain, touch, and itch^16^. We next used this resource to benchmark dIs derived using a new human NMP-based differentiation protocol designed to recapitulation *in vivo* development. To generate dIs across the entire anterior-posterior axis of the spinal cord, we systematically modulated two parameters previously implicated in posterior patterning^18, 55^: the duration of NMPs culture and exposure to GDF11, which regulates posterior *HOX* gene expression^56^. Comparison with the reference atlas, revealed that *in vitro*-derived dIs are transcriptionally indistinguishable from their endogenous counterparts. Moreover, further analysis of the dI4/dI5 subtypes identified many of the same sensory modalities, some with conserved molecular signatures. Notably, protein interaction network analysis revealed enrichment for genes with allele variants that highly associated with autism spectrum disorder (ASD), raising the possibility that spinal-cord-intrinsic mechanisms contribute to ASD-associated phenotypes.

## Results

### Creation of human fetal scRNA-Seq atlas yields distinct separation of spinal cord cell types

**Extended Data Figure 1:**
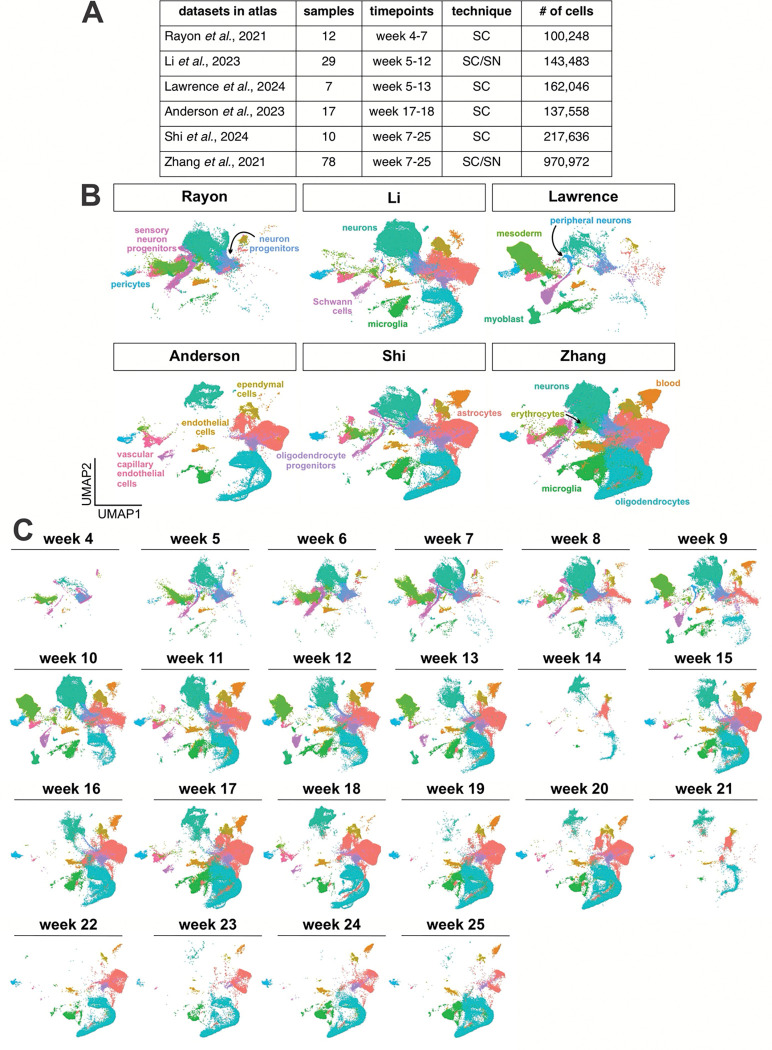
Annotation of different data sets by week in the complete *in vivo* atlas (A) Table showing summary statistics of the 6 datasets used in generation of the atlas. (B) These datasets each contain different mixtures of cell types, with some containing substantially more neuronal lineage cells than others (C) UMAPs split by week, showing progression of cell type differentiation over time, with early expansion of neurons and mesoderm, followed by astrocytes and oligodendrocytes

**Figure 1:**
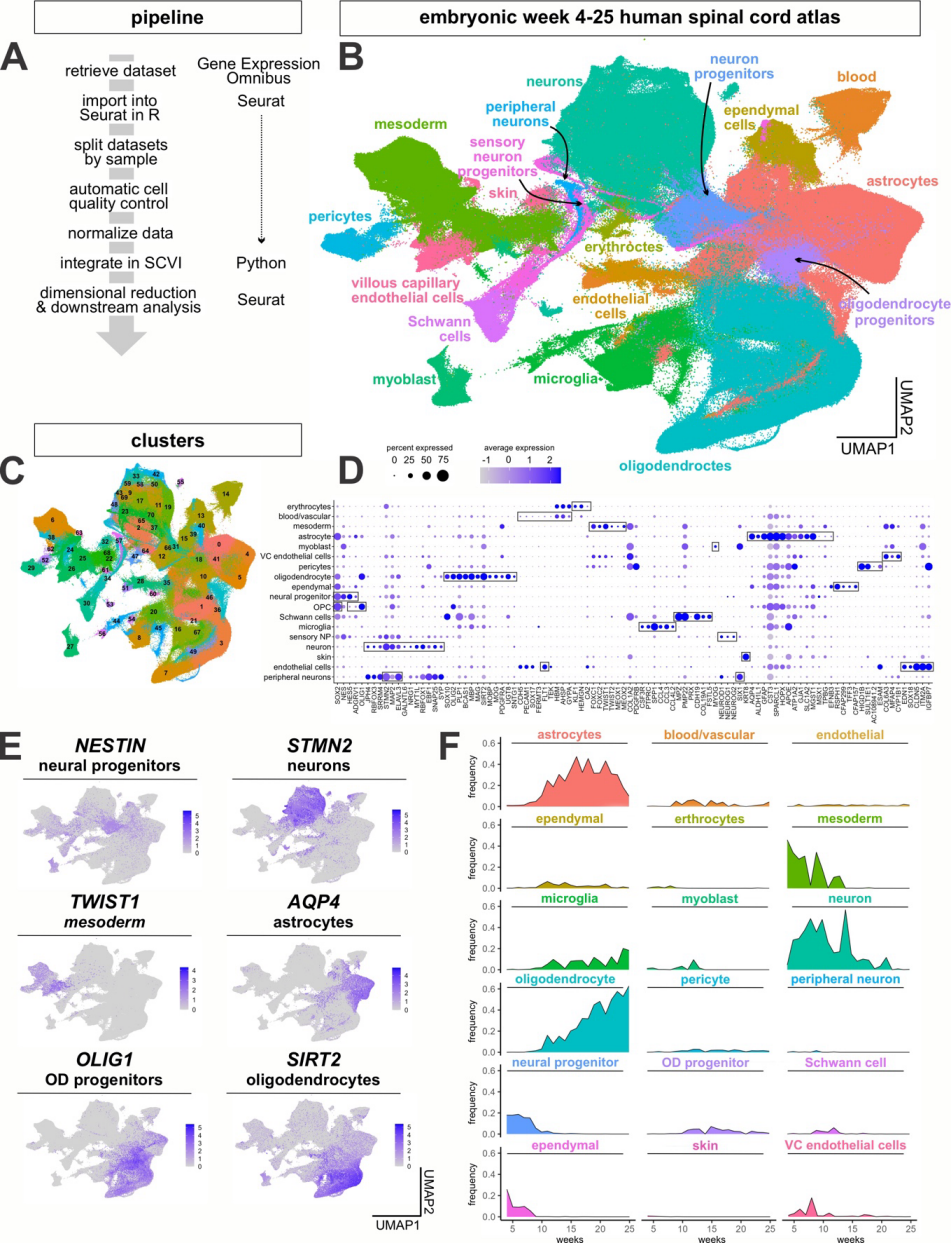
Construction of an *in vivo* human embryonic spinal cord scRNASeq reference atlas (A) Overview of the analysis pipeline for the single-cell transcriptomic data. (B) The *in vivo* human embryonic spinal cord single cell atlas. Integration of 1.7 million cells from 192 samples spanning gestational weeks 4 to 25 reveals 18 broad categories of cell types present in the spinal cord and surrounding tissues. (C, D) Unsupervised clustering yielded 71 distinct transcriptional clusters (C) that could be grouped into 18 cell types (B), based on their expression of canonical marker genes (boxed genes, D). (E) UMAPs depicting the expression of representative marker genes in the *in vivo* human embryonic spinal cord single-cell atlas. (F) Comparison of the frequency by which the 18 cell types emerge over the timeline of the atlas demonstrates the early proliferation of mesodermal and neuronal cell types, with the later proliferation of astrocytes and oligodendrocytes. The apparent increase in neurons at week 14 likely reflects that all W14 datasets consist of single nuclei samples, whereas datasets from other time points are predominantly single-cell samples.

To create a reference atlas for the developing human spinal cord, we compiled transcriptomic data that spans fetal development (Fig. 1b). We integrated 192 publicly available sc and single nuclei (sn) RNA-Seq samples from 6 studies^28, 50–54^ (Extended Data Fig. 1a, b), to create an atlas of 1.7 million cells spanning gestational weeks 4 to 25 (Extended Data Fig. 1c). We established the following pipeline to integrate such a large number of samples (Fig. 1a): datasets were initially preprocessed for quality control in Seurat^57^ in R, before combining the samples using the machine learning pipeline SCVI^58^ in Python. The resulting dimensional space was then transferred back to Seurat for projection into UMAP space and cell type labelling (see Materials and Methods for a complete description of the computational pipeline).

Using a curated list of cell markers (Fig. 1d), the sctype package^59^ was used to classify the 71 clusters (Fig. 1c) into 18 cell types of the spinal cord. These cell types arise from either ectodermal or mesodermal derivatives. Their proportions vary in the different studies (Extended Data Fig. 1b), which reflects both the time period covered by each dataset, as well as any differences in dissection and processing methods. The cell type classification method uses the combinatorial expression of multiple genes to assign cellular identity (Fig. 1d), but we also found that canonical single-gene markers reliably segregate the different cell types (Fig. 1e). As a validation of the integrity of the atlas, we assessed how the proportion of cell types change from weeks 4 to 25 (Fig. 1f). The cellular proportions recapitulate the expected developmental ontogeny^60, 61^, showing the highest numbers of progenitors and mesoderm at weeks 4/5, followed by the birth of neurons, and then increasing numbers of astrocytes, and then oligodendrocytes (Fig. 1f).

### Analysis of the neuronal subset in the atlas identifies a dramatic expansion of dI4/5 populations

**Figure 2:**
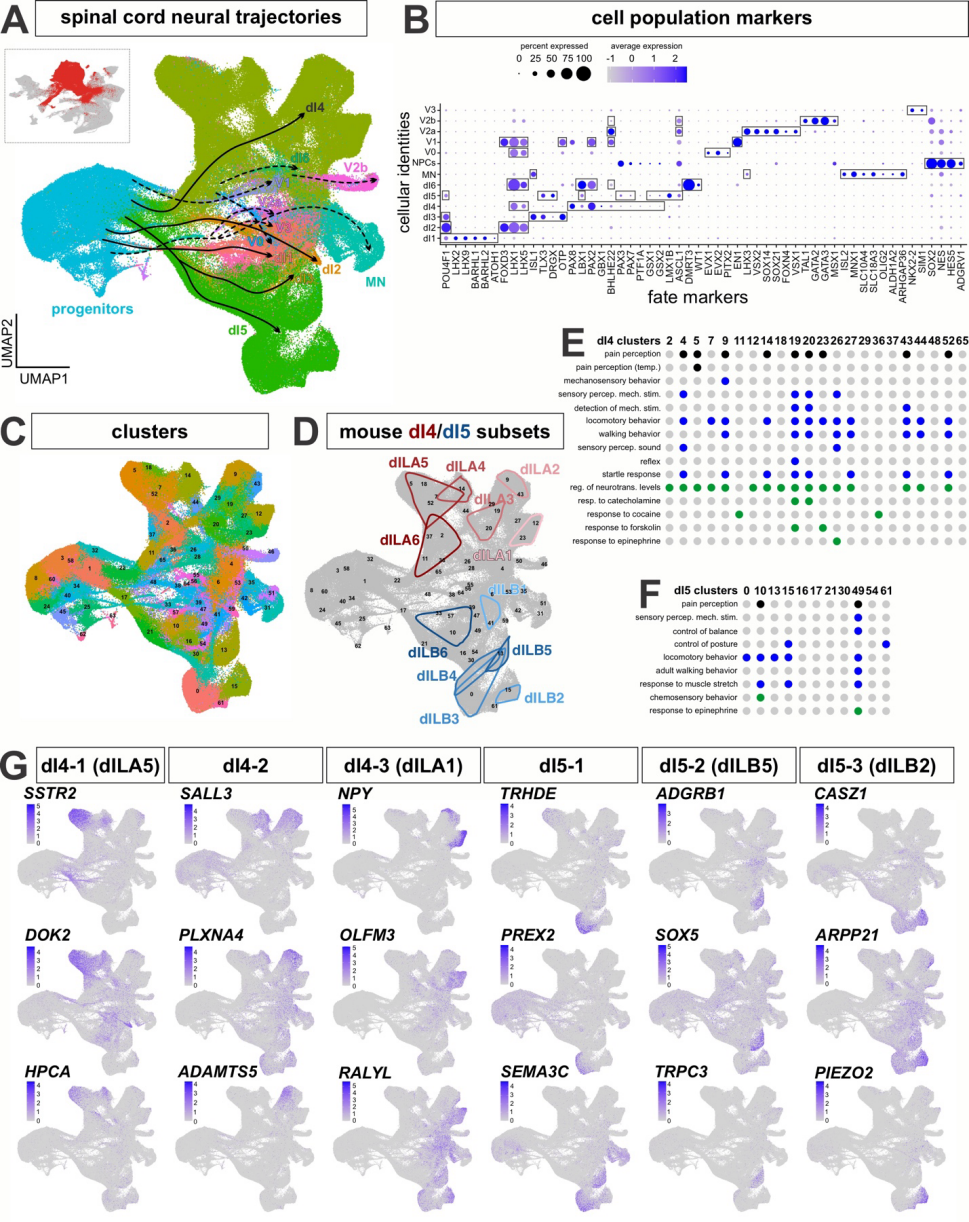
Analysis of dI lineages within the *in vivo* human reference atlas (A) ~300,000 neuronal lineage cells were extracted from the overall atlas (inset) and then plotted as 2d UMAP reduction. Labeling cells taken from across the complete timeline with the AUCell classification system identified all the dorsal (dI1-dI6) and ventral (v0-v3, MNs) neural identities in the spinal cord and revealed a vast expansion of the dI4 and dI5 lineages. The differentiation trajectories were hand annotated using gene expression patterns. (B) Dotplot of genes used for classification of each cell type shows expression restricted to expected cell types. (C) Clustering of the neuronal atlas yielded 66 clusters. (D) The expansion of human dI4/dI5 in comparison was assessed in comparison to an embryonic mouse spinal cord single cell atlas^63^. In the mouse study, dI4/dI5 subtypes were designed as late born dILs, dILA1–6 (Extended Data Figure 4A) and dILB1–6 (Extended Data Figure 4B). (E, F) Gene Ontology (GO) enrichment analysis was performed on significantly upregulated genes (avg_log2FC > 0.25) for all clusters identified as either dI4 or dI5 subtypes. Dot plots of GO Biological Process terms found across dI4 (E) and dI5 (F) clusters include various behavioral functions, largely aligning with the canonical roles dI4 and dI5s play in processing sensory information. Black dots identify GO terms related to the sensory perception of pain and temperature; blue dots signify GO terms from different sensory modalities including touch, sound, movement and balance; green dots represent GO terms associated with a response to chemical stimulants. (G) Unique gene markers were identified late-stage subsets of dI4 (dI4–1, dI4–2 and dI4–3) and dI5 (dI5–1, dI5–2 and dI5–3) to further demarcate additional distinct cellular subtypes within the dI4 and dI5 lineages. These genes were selected for their specificity to the cluster, with low to no expression in other clusters.

We next subsetted the dataset to extract the cells classified as neuron progenitors, sensory neuron progenitors, neurons, and peripheral neurons. After a further round of integration and subsetting to specifically retain the spinal neural trajectories, we obtained a neuronal-specific atlas containing 308,947 cells, including 56,674 progenitors) (Fig. 2a). Using the AUCell tool^62^ (Fig. 2b) and a custom gene list (Extended data Table1 and 2) to classify cell types, we were able to designate all of the cells according to their dorsal or ventral spinal neuronal identities (Fig. 2a). The atlas identifies a progenitor domain, and 12 distinct spinal neural trajectories (dorsal: dI1-dI6; ventral: v0, v1, v2a/b, MNs and v3). Each of these cell types show distinct expression of their canonical marker genes with little overlap between unrelated cell types (Extended Data Fig. 3a, b). These cell types can be further subdivided into 66 clusters (Fig. 2c), of which ~50 clusters correspond to the neuronal identities.

**Extended Data Figure 2:**
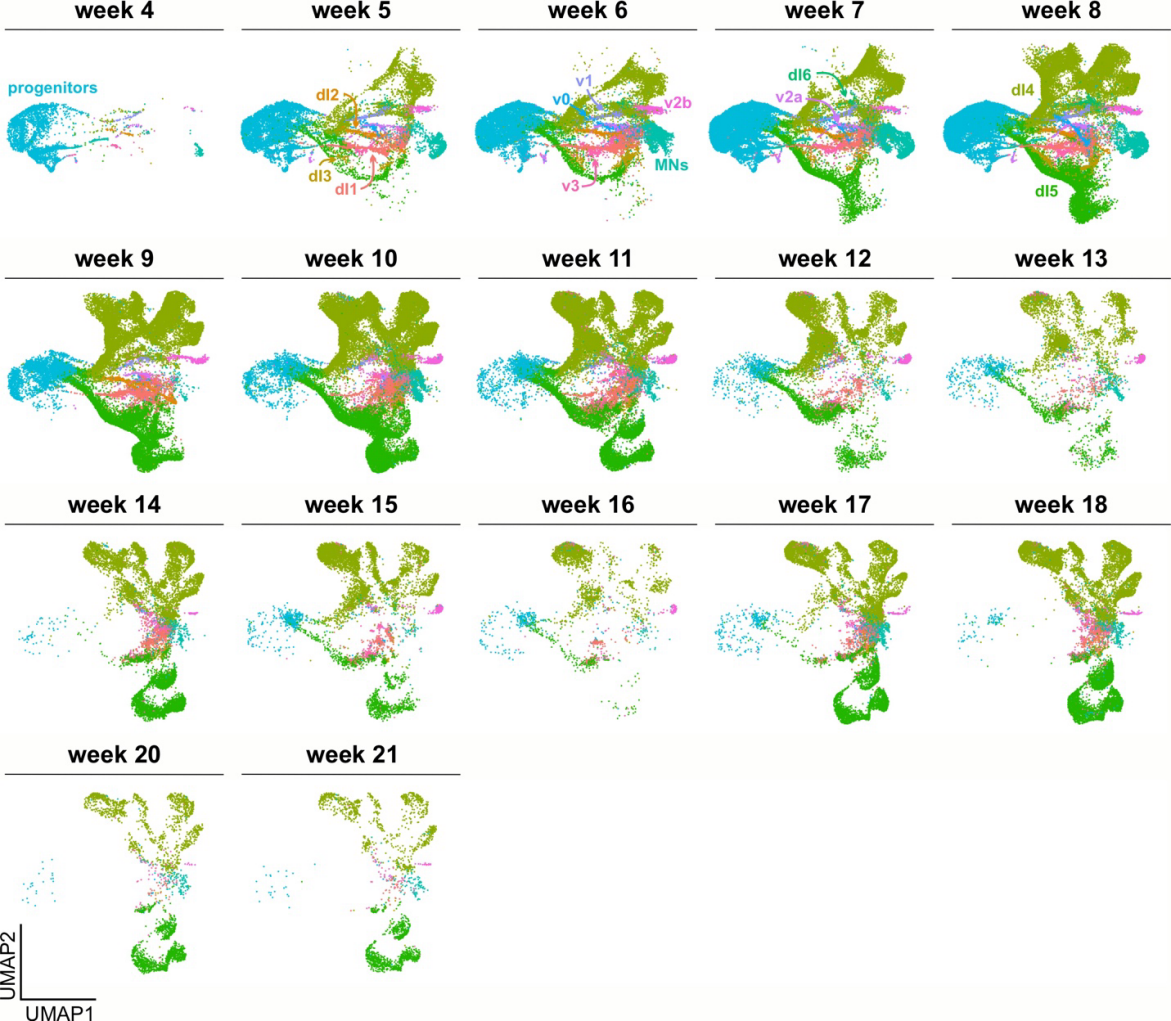
Annotation of cell types in the neuronal *in vivo* atlas by week Breakout of neuronal atlas cell types by week of sample. Early weeks contain more progenitors and earlier stages of neuronal types. Later weeks contain mostly cells in the extremities of the UMAPs, many of these cell types are cells that contain gene expression overlapping with dILs.

The dI4s (123,620 cells, found predominately in 22 clusters) and dI5s (68,603 cells, 11 clusters) are by far the largest populations of neurons (Fig. 2a). dI4s and dI5s arise at week 5 of gestation, together with the other neuronal lineages. They then undergo a major period of expansion from week 7–11 (Extended Data Fig. 2) such that they ultimately make up 40% (~50% if the neural progenitors are excluded) and 22% (30%) of the neuronal subset respectively. To determine the identities of potential dI4/5 subtypes, we first assessed how the human dI4/dI5s align with a recently published embryonic mouse spinal cord single cell atlas^63^. This mouse atlas builds on previous developmental studies in mouse and chicken *in vivo* that defined a late born population of dIs, called the dILs, that differentiate from the same region as the dI4/dI5s *in vivo*^64^. The dILs exist as two subpopulations, dILA and dILB, which have overlapping molecular signatures with dI4s and dI5s respectively. The mouse populations of dILA/dILB are also massive and can be further subdivided into 12 subpopulations^63^ (dILA1–6; dILB1–6). We used AUCell to calculate module scores for each of the dIL subtype identities and then computationally mapped the top 50 cells in each dIL subtype onto the human atlas (Fig. 1d; Extended Data Fig. 4a, b). As further validation of the human atlas, the mouse dILA/dILB subpopulations map onto the human dI4/dI5 regions in discreet clusters, suggesting that these subpopulations are evolutionarily conserved. The general temporal topology of the clusters is also conserved between mouse and human, following a similar progression away from the progenitor domain (Fig. 2d). The transcriptomic resemblance between the dILA/dILB and dI4/dI5 populations suggests they may represent the same cell type. Of the 33 human dI4/dI5 clusters, ~24 clusters overlap between mouse and human. Moreover, while all of the mouse modules are accounted for, ~9 clusters are unique to the human atlas, suggesting the existence of human specific functions.

### Analysis of dI4/dI5 subtypes reveal multiple sensory function signatures

We next assessed whether the gene expression profile in each cluster can be used to indicate the function of the dI4/dI5 subtypes. We performed marker gene analysis on each of the dI4/dI5 subclusters followed by gene ontology. Cross comparison of the resulting GO Biological Process terms among dI4 and dI5 clusters identified both shared and unique behavioral functions (Fig 2e, f). Shared dI4/dI5 functions include pain perception, the detection of mechanical stimulus (i.e. touch), and regulation of movement, while dI4 subtypes uniquely detect temperature (cluster 5), control the startle response and the sensory perception of sound (cluster 4 and 26). We additionally found dI4 subtypes (clusters 11 and 36) that respond to cocaine, supporting our previous observation that embryonic dI4/dI5s may be responsive to psychoactive compounds^44^.

We also sought to identify marker genes as another method of identifying dI4/dI5 subtypes (Fig. 2i). By manually curating marker gene lists, we thereby identified functionally relevant gene associations for several clusters. These genes include well-known neuronal associated genes such as *SSTR2* (cluster 5, which overlaps with mouse dILA5) which modulates neurotransmission^65^ and itch^66^, and *NPY* (cluster 12, which overlap with mouse dILA1/2), a peptide neurotransmitter that can inhibit pain perception^67^. Other functionally relative genes include *PIEZO2* (cluster 15, which overlaps with mouse dILB2) the mechanosensitive ion channel that senses light touch, proprioception, and mechanical pain^68^. Taken together, these findings suggests that that dI4/dI5 lineage undergoes significant diversification towards specialized subtypes.

### Deriving human NMPs from along the anterior-posterior axis in vitro

Our longstanding scientific interest is to generate *bona fide* dIs from human pluripotent stem cells that represent the major axial levels of the spinal cord, i.e., cervical, thoracic, lumbar, and sacral, to work towards a cellular replacement therapy for transplantation approaches. Our previous studies with mouse ESCs^44^ found that generating dIs through a NMP intermediate accurately reproduces the full diversity of dIs found *in vivo*, producing cells that are transcriptionally indistinguishable from their endogenous counterparts. Here, we adapted this NMP-based protocol for human ESCs and additionally examined how both extended culture duration and GDF11 treatment^18, 55, 69^ influence dI patterning along the anterior-posterior and dorsal-ventral axes of the spinal cord.

**Figure 3:**
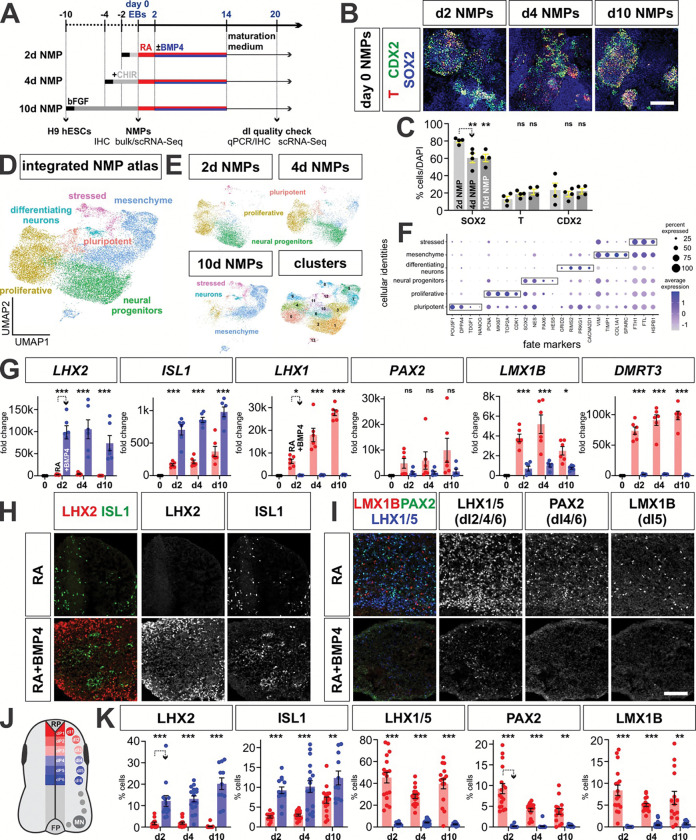
Specification of dI identity in the human RA±BMP4 NMP protocol (A) Overview of the experimental timeline/workflow for the RA±BMP4 NMP protocols. H9 hESCs were cultured with bFGF/CHIR to induce day (d) 2, 4 and 10 NMPs under 2-dimensional (2D) culture conditions. At day 0, NMPs were sent for both bulk and single cell (sc) RNA sequencing (RNA-Seq) for transcriptional analyses. The NMPs were then converted into 3-dimensional (3D) embryoid bodies (day 0 EBs) and then treated with a 2-day pulse of RA, followed by RA+BMP4 for 12 days to induce dorsal patterning. qPCR and immunohistochemical (IHC) quality checks were performed on day 20. (B, C) IHC analysis of d2 d4 and d10 day 0 NMPs with antibodies directed against T (BRACHYURY, red), CDX2 (green) and SOX2 (blue). Quantification suggests that the number of NMPs is equivalent across the three conditions. (D) UMAP projection of the complete complement of day 0 NMPs, i.e. 2d, 4d and 10d, demonstrates that the cells segregate into neural progenitor and mesenchymal progenitor populations. (E) UMAPs split by culture duration prior to NMP induction depict a progressive shift from neural progenitor enrichment in 2d and 4d NMPs towards mesenchymal identity in 10d NMPs. (F) Dot plot of different NMP fates showing the expression levels of marker genes. (G-I, K) Both qPCR (G) and IHC (H, I, K) analyses of canonical dI markers in day 20 EBs, demonstrate that the RA and RA+BMP4 protocols, using d2, d4 and d10 NMPs, generate the predicted complement of dIs, i.e., RA→dI4, dI5, dI6 and RA+BMP4 → dI1 and dI3. (J) Schematic representation of the developing spinal cord *in vivo*, depicting the location of the six populations of dorsal progenitors (dP) and dorsal interneurons (dIs). Scale bar: 100μm (B); 75μm (H, I) Probability of similarity between control and experimental groups: *= p < 0.05, **p<0.005 *** p<0.0005; two-way ANOVA.

**Figure 4:**
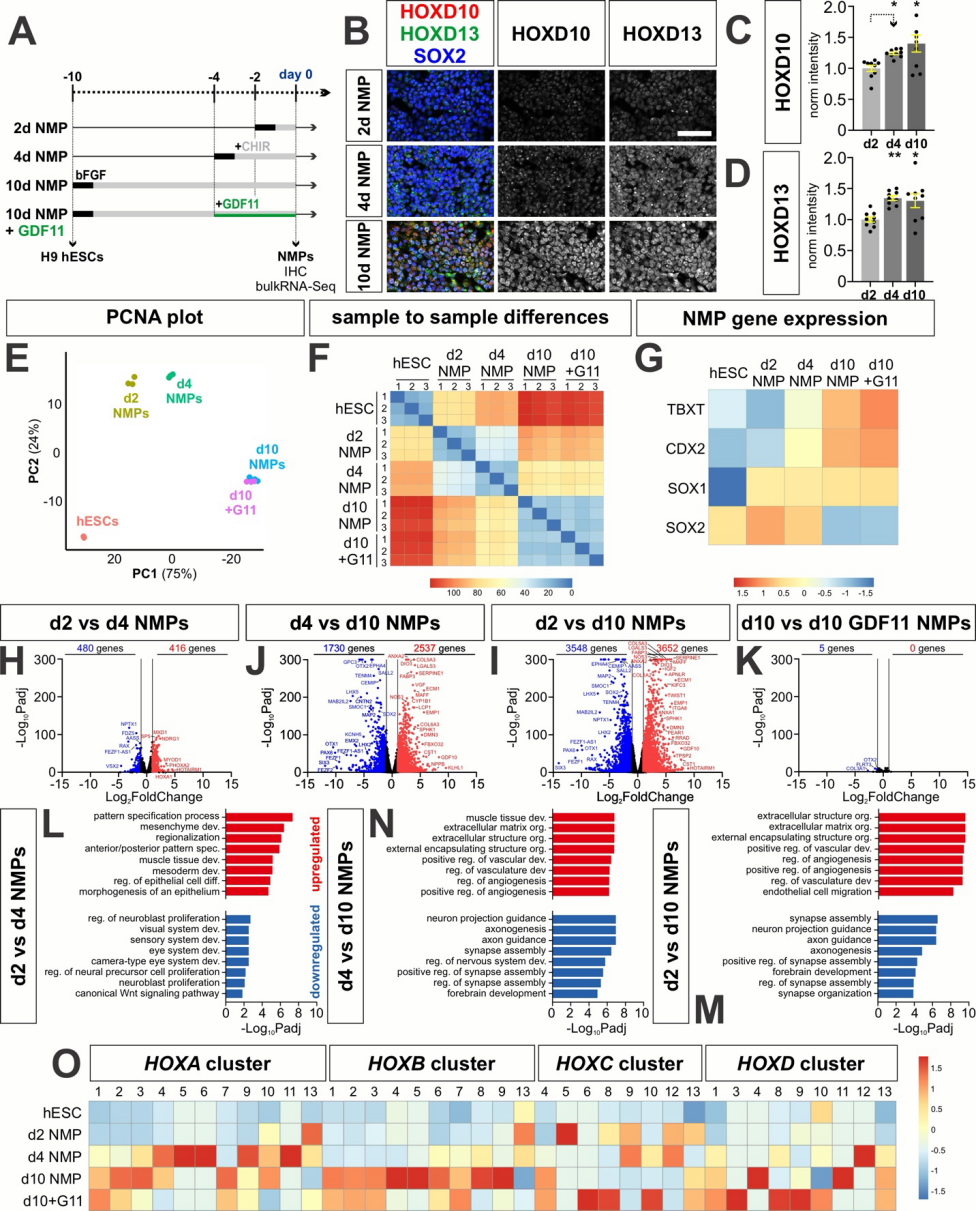
Assessing the effect of culture duration on NMP identity along the anterior-posterior axis (A) Overview of the experimental timeline/workflow for the RA±BMP4 NMP protocols. (B -D) IHC analyses of NMPs immediately before EB formation also suggests that prolonging the culture time progressively posteriorizes NMPs. Two posterior HOX genes - HOXD10, and HOXD13 - have significantly higher expression in d10 NMPs, compared to d2 NMPs. SOX2 levels are ~constant across the d2, d4 and d10 NMP conditions, indicating that the NMP identity is maintained. (E) Principal-component analysis (PCA) of bulk RNA-seq expression profiles from hESCs, d2, d4, d10, and d10+GDF11 NMPs shows that samples segregate along PC1 (75%) according to the duration of NMP induction, suggesting culture time is the primary driver of transcriptional variation. (F) Heatmap of pairwise Euclidean distances between samples. hESCs are most dissimilar from d10 and d10 + GDF11 NMPs, while d10 and d10+GDF11 samples are highly transcriptionally similar. (H-K) Volcano plots comparing the differentially expressed genes (DEGs) in the d2, d4 and d10 NMP cultures (|log_2_ fold change| ≥ 1, Benjamini-Hochberg-adjusted p < 0.05). The magnitude and number of transcriptional differences increase with greater separation in induction time, with the largest number of DEGs observed between d2 and d10 NMPs, and minimal differences between d10 and d10+GDF11 NMPs, indicating largely shared transcriptional states. (L-M) Gene Ontology (GO) enrichment analysis of significantly upregulated and downregulated genes in contrasts. Genes upregulated in d4 NMPs are enriched for patterning and morphogenetic processes, while d10 NMPs show strong enrichment for extracellular matrix organization and vasculature-associated pathways. Downregulated gene sets suggest that forebrain-associated transcriptional programs are progressive reduced with increased culture time. (O) Heatmap of average *HOX* gene expression across conditions, scaled per gene. Robust *HOX* expression emerges in d4 NMPs, predominantly in the HOXA cluster. d10 NMPs show strong *HOXB* and *HOXD* gene expression, while the addition of GDF11 increases expression in the *HOXC* and *HOXD* clusters, suggesting that prolonged culture duration and GDF11 exposure play a role modulating axial identity through differential activation of *HOX* clusters. Scale bar: 20μm Probability of similarity: *= p < 0.05, **p<0.005; two-way ANOVA.

Human NMPs were generated by exposing the H9 stem cell line to bFGF and CHIR, a Wnt agonist^44, 70^ (Fig. 3a). To investigate whether NMPs can be generated along the anterior-posterior axes of the spinal cord, we assessed the effect of extending the time of the NMPs in culture, which has previously been shown to result in the sequential induction of the HOX genes^18^. In this model, extending the duration of FGF/CHIR exposure drives anterior-to-posterior specification *in vitro*. hESCs were treated for 2, 4, or 10 days in FGF/CHIR to generate 2d, 4d and 10d NMPs, respectively (Fig. 3a, b). In all conditions after bFGF/CHIR treatment (“day 0” in Fig. 3a), similar proportions of cells were labeled in immunohistochemical (IHC) studies, using antibodies for the canonical NMP markers (SOX2^+^/T^+^/CDX2^+^; Fig. 3b, c). We also performed single cell (sc) (Fig. 3d–f) and bulk (Fig.4e–o) RNA-Seq at day 0. Using marker gene expression to classify cell types (Fig. 3f), bipotential neural–mesenchymal identities were observed in scRNA-Seq transcriptomic profiling (Fig. 3d), although both the sc- and bulk-RNASeq analyses suggest the clusters become progressively more biased towards mesoderm derivatives as time progresses (Fig. 3e, Fig. 4g, h–j, l–n). Both volcano and Gene Ontology (GO) term analyses from the bulk-RNASeq data additionally show that forebrain signatures are progressively reduced the longer NMPs spend in culture (Fig. 4g, h–j, l–n). Taken together, these studies suggest the successful derivation of bipotential NMPs (Fig. 3b, d).

We next determined whether extending the duration of FGF/CHIR treatment induces NMPs with more posterior HOX identities (Fig 4a). As a first approach, we assessed the expression of posterior HOXD genes in a IHC analysis performed at day 0 (Fig. 4a). The levels of HOXD10 and HOXD13 are undetectable in 2d NMPs (Fig. 4c, d) and steadily increase in d4 and d10 NMPs (Fig. 4c, d), suggesting that posterior HOX genes are progressively induced in d4/d10 NMPs, compared to d2 NMPs (Fig. 4c). To comprehensively identify axial identities by assessing the expression of all *HOX* genes, we further analyzed the day 0 bulk RNA-Seq data set. Principal-component analysis (PCA) of this data revealed that PC1 and PC2 contribute 99% of the variation in the data and successfully separate the hESCs, d2, d4 and d10 NMPs (Fig. 4e). We also assessed the Euclidean distances between the gene expression profiles, where increased distance (red) between samples indicates dissimilarity^71^. This analysis suggests that the d2, d4 and d10 NMPs become progressively more distinct from hESCs (Fig. 4f). Similarly, our volcano analyses show that the number of differentially expressed genes increases as the NMPs progress from d2 to d10 (Fig. 4h–i). However, the volcano and GO term analyses also reveal that the differentially expressed genes appear to be functionally consistent, i.e. progressing towards a mesodermal identity and away from anterior neural signatures (Fig. 4g, h–j, l–n). Supporting the observation that the NMPs become progressively posteriorized, HOX gene expression increases the longer NMPs spend in culture (Fig. 4o). Thus, the 2d NMPs have generally low levels of HOX gene expression, with only *HOXC*5 showing high expression, consistent with a cervical identity^72^. In contrast, *HOX* gene expression is upregulated in 4d NMPs, with high levels of expression in the *HOXA* cluster (Fig. 4o). This trend continues with 10d NMP, which have widespread high levels of *HOX* gene expression in the *HOXA*, *HOXB* and *HOXD* clusters, including the posterior HOX genes, such as *HOXA10*, *HOXD11* and *HOXD13*. Together, these data demonstrate that extending FGF/CHIR treatment progressively directs posteriorization of human NMPs.

### Human NMPs can be directed towards the complete complement of dIs

We next asked whether d2, d4 and d10 NMPs can generate the complete range of dI subtypes (Fig. 3j). NMPs were permitted to form three-dimensional embryoid bodies (EBs) at day 0^73^, and then neuralized with retinoic acid (RA) until day 14 (Fig. 3a). qPCR and IHC analyses performed at day 20 demonstrated that the RA protocol robustly and consistently generates the full complement of intermediate dIs, i.e., dI4, dI5, dI6, from d2 d4 and d10 human NMPs (Fig. 3g–k, Extended Data 4e) as shown previously for mouse NMPs^44^. These mouse studies also showed that adding BMP4 after RA directs the dorsal-most dIs, i.e., dI1, dI2, dI3^44^. We optimized the timing of BMP4 addition for human d4/d10 NMPs and thereby identified day 2–14 as the most effective period for BMP4 treatment (Fig. 3a, Extended Data 4a–d). qPCR and IHC analyses demonstrated that the RA+BMP4 protocol robustly and consistently generates significant numbers of dI1s and dI3s from d2 d4 and d10 human NMPs (Fig. 3g–k, Extended Data 4e). Our subsequent scRNA-seq analysis (Fig. 6a) indicates that we also generate dI2s, which were not detected with our qPCR or IHC assays. Collectively, these results establish human NMP differentiation protocols which recapitulate *in vivo* HOX patterning along the anterior posterior axis and produces the full complement of dI diversity.

### GDF11 modulates of dI patterning in stem cell-derived dorsal progenitors

**Figure 5:**
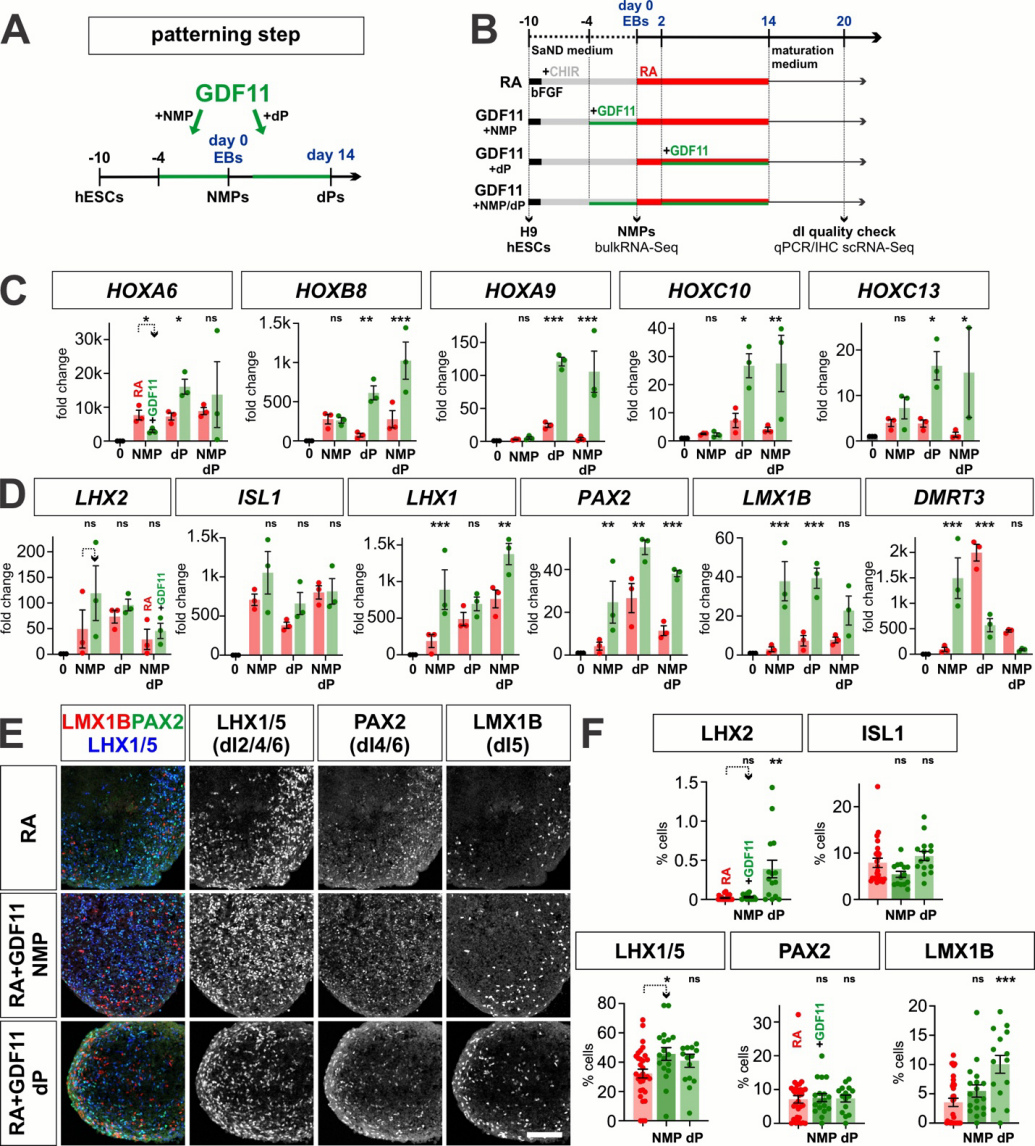
Assessing the effect of GDF11 treatment on both dorsal-ventral and anterior-posterior identity (A, B) Overview of the experimental timeline/workflow for the RA±GDF11 NMP protocols. The effect of GDF11 treatment was assessed on NMP patterning, dP patterning and both NMP and dP patterning (A). (C) qPCR analyses of a range of *HOX* gene expression suggests that GDF11 addition at the dP stage is the most effective at driving the expression of the posterior most HOX genes (*HOXA9*, *HOXC10* and *HOXC13)*. (D-F) qPCR analyses of day 20 EBs (D), suggest that the addition of GDF11 at either the NMP or dP stage significantly increases expression of *PAX2* and *LMX1B*, the canonical genes that mark the dI4/dI5 identities. However, the IHC analysis suggest the effect of GDF7 on protein levels is more subtle, with the most significant effect observed when GDF11 added at the dP stage on dI5 identities. Scale bar: 75μm Probability of similarity between control and experimental groups: *= p < 0.05, **p<0.005 *** p<0.0005; two-way ANOVA

GDF11 regulates posterior HOX patterning in the neural tube^56^ and has been shown to induce posterior HOX genes in differentiating hESC-derived NMPs^18, 55^. Additionally, *Gdf11* is expressed in the mouse intermediate spinal cord during dI fate determination ^69^. Thus, we assessed whether GDF11 has a role patterning either NMPs or dorsal progenitors (dPs) in our protocol, directing posteriorization and/or the specification of dI fate(s). GDF11 was added to the 10d NMP protocol for [1] 6 days at the NMP stage from day −4 to 0 (Fig. 4a, 5a), [2] 12 days at the dP stage from day 2–14 after EB formation, and [3] at both the NMP and dP stage (Fig. 5a, b). HOX gene expression profiles were analyzed by bulk RNA-Seq at day 0 (Fig. 4o), and by qPCR at day 20, while the dI identities were assessed in day 20 EBs.

As described above, 10d NMPs express high levels of *HOX* gene expression (Fig. 4o). The addition of GDF11 for 6 days has surprisingly minimal effects on the identity of NMPs. The d10 and d10+GDF11 NMPs map on top of each other in the PCA plot (Fig. 4e), a volcano analysis reveals almost very few differentially expressed genes (Fig. 4k). However, the bulk RNA-Seq suggests that the addition of GDF11 may alter the cadence of *HOX* gene expression. HOX genes are still widely expressed by 10d+GDF11 NMPs, with highest expression in *HOXC* and *HOXD* cluster, while 10d NMPs showed higher *HOXB* and *HOXD* expression levels (Fig. 4o). However, qPCR profiling of *HOX* gene expression at day 20 suggests that the addition of GDF11 at NMP does not have lasting effect on posteriorization. Rather, further posteriorization is achieved by adding GDF11 at the dP stage, which further drives the expression of *HOXA9*, *HOXC10* and *HOXA13*, consistent with a lumbosacral identity (Fig. 5c).

GDF11 treatment also directs dI patterning. Although GDF11 addition results in minimal alterations in the specification of the dorsal-most dIs, dI1–dI3 (Fig. 5d, f), it induces dramatic increases in *PAX2* and *LMX1B* expression, present in dI4s and dI5s respectively, compared to RA alone (Fig. 5d). Consistent with this result, there is a 2-fold increase in the number of LMX1B^+^ dI5s after GDF11 treatment, compared to RA alone (Fig. 5e,f). GDF11 addition at either the NMP or dP stage increases gene expression; however, only treatment at the dP stage results in an increase in LMX1B^+^ dI5s, suggesting that GDF11 acts in a temporally restricted manner to specify dI identity (Fig. 5e,f). For all conditions, there appears to be no synergistic effect of adding GDF11 at both the NMP and dP stages. GDF11 also appears to have minimal effect on *DMRT3* expression, the canonical marker for dI6s (Fig. 5d), and generally acts to suppress the effect of BMP4 addition (Extended Data Fig. 5). Taken together, these studies suggest GDF11 is most effective during dP specification, where it drives posteriorization and specifically reinforces dI5 identity in differentiating dPs. GDF11 may thus serve as an additional inductive cue that can promote specific dI subtypes *in vitro.*

### Comparison of in vitro-derived dIs with the in vivo human embryonic spinal cord atlas

**Figure 6:**
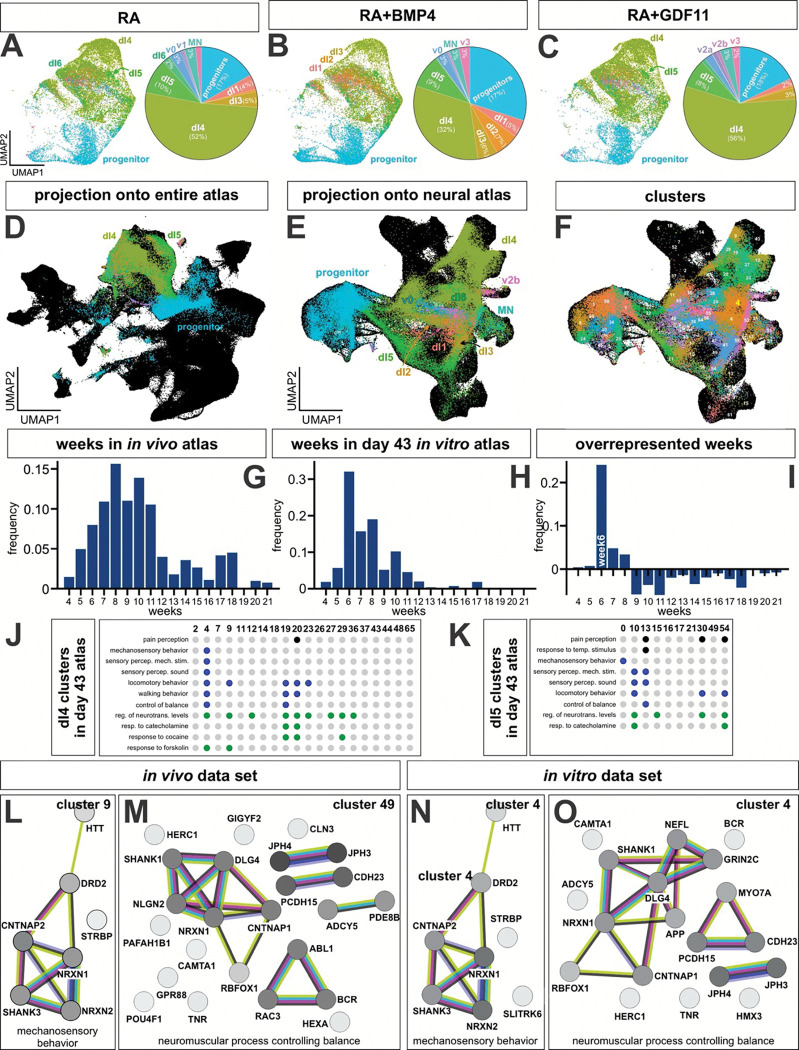
Comparison of the day 43 *in vitro* dataset with the human *in vivo* reference atlas reveals ASD signatures (A-C) UMAPs of day 43 *in vitr*o-derived cells generated using the RA, RA+BMP4, and RA+GDF11 (NMP) protocols, using neural spinal cord cell-type identity transferred from the *in vivo* neuronal atlas (Fig, 2a). The pie charts summarize the proportions of neuronal cells generated by each protocol. The RA conditions predominantly yield dI4-dI6 identities, whereas RA+BMP4 shifts patterning toward dI1-dI3 populations. Addition of GDF11 prior to NMP induction does not alter resultant cell-type composition compared to RA alone. (D). Projection of the day 43 *in vitro* cells onto the complete *in vivo* spinal cord reference (shown as a black silhouette). I*n vitro* neural progenitors and neurons map directly onto their *in vivo* counterparts, selectively recapitulating endogenous spinal neural transcriptional identities. (E, F) Projection of day 743 *in vitro-*derived cells onto the neuronal subset of the *in vivo* spinal cord atlas. The *in vitro* dIs map along the correct lineage trajectories (E) suggesting they are transcriptionally indistinguishable from endogenous neurons. The *in vitro* dIs are colored by transferred neuronal cell-type identity (E) or atlas cluster identity (F). (G-I) The day 43 *in vitro* datasets (G) align most closely with week 6 samples in the *in vivo* reference atlas (H). Using the relative frequency of the developmental weeks in the *in vivo* atlas as a reference, we assessed for overrepresentation and found that week 6 profiles were enriched in the *in vitro* dataset compared with the *in vivo* atlas (I). (J, K) Gene Ontology (GO) enrichment analysis was performed on significantly upregulated genes (avg_log2FC > 0.25) for any stem cell derived clusters assigned to a dI4 or dI5 atlas cluster, as performed previously for the *in vivo* dI4/dI5 clusters. Dot plots of enriched GO Biological Process terms in the dI4 (J) and dI5 (K) clusters include behavioral categories that overlap with those observed in the in vivo datasets (Fig. 2e, f). Black dots are related to the sensory perception of pain and temperature, blue dots correspond to sensory modalities including touch, sound, movement and balance, while green dots represent a response to chemical stimulants. (L-O) To assess the extent to which *in vitro* and *in vivo* dIs may be functionally similar, the genes making up each GO term were subjected to a STRING analysis, thereby identifying the protein interaction networks that encode specific sensory behavioral modalities. These networks include a mechanosensory circuit derived from *in vivo* cluster 9 (L), as well as a balance regulating circuit derived from *in vivo* cluster 49 (M), include proteins with variants that was strongly associated with autism spectrum disorder: NLGN2, NRXN1, SHANK1/3, DLG4 and CNTNAP2. These circuits were recapitulated in the *in vitro* cluster 4 mechanosensory behavior protein interaction network (N) and the *in vitro* cluster 4 balance regulating protein interaction network (O). For STRING networks (L-O), nodes represent proteins, edges represent protein-protein associations. Edge color indicates known interactions; experimentally determined (purple), from curated databases (teal), predicted interactions; gene neighborhood (green), gene fusions (red), gene co-occurrence (blue) and others; textmining (yellow), co-expression (black), protein homology (periwinkle)

To begin to determine how the cells mature in culture, neurons derived from the RA, RA+BMP4 and RA+GDF11 (NMP only) protocols were cultured to day 43 and then assessed for their cellular identity by scRNA-Seq (Fig. 6a–c). Confirming previous results, there was very little impact of GDF11 added at the NMP stage, the UMAPs of RA and RA+GDF11 are essentially indistinguishable. Both data sets make large numbers of dI4 (50%) and dI5s (~10%), while the RA condition makes ~2% dI6s. The addition of BMP4 is effective in reducing the number of dI4s, and making increased numbers of the dorsal most dIs, most notably specifically dI2s. Interestingly, ventral neurons, i.e. v0, v1, v2a, v2b, v3, and motor neurons (MNs) are also found in small percentages in all conditions, suggesting that the EBs generate their own patterning centers which further recapitulate the entire spinal cord.

To assess how closely the day 43 *in vitro*–derived dIs resemble their in vivo counterparts, we used scVI and scArches^74^ to project the *in vitro* single-cell datasets onto both the full reference atlas (Fig. 1b) and the neuronal-specific atlas (Fig. 2a). When given the choice of the spinal identities in the complete atlas, the *in vitro* derived neurons specifically aligned with the neuronal identities in the *in vivo* atlas, rather than the non-neuronal cell types (Fig. 6d). Transferring cell identity labels from the *in vivo* atlas to the *in vitro* datasets, revealed a marked preference for generation of dorsal spinal identities. When mapped onto the neuronal-specific atlas, the *in vitro*-derived dIs map along the corresponding *in vivo* lineage trajectories (Fig. 2a, 6e). Taken together, this data suggests that the stem-cell derived dIs are transcriptionally indistinguishable from their endogenous counterparts.

However, the *in vitro* dataset overlaps primarily with the regions present in earliest time points in the atlas, i.e., weeks 4–7 and is notably absent from the dI4/dI5s that are specified from week 8 onward (Fig. 6f, Extended Data Fig. 2). We compared the age of day 43 data set to the reference atlas by training a classifier on the week metadata in the scVI space of the neural atlas and transferring these labels to our *in vitro* dataset. Comparing the distribution of cells per week in the reference atlas (Fig. 6g) with that of the day 43 dataset (Fig. 6h), identified week 6 as the most overrepresented time point in the *in vitro* dataset, consistent with their chronological age in culture. Thus, the *in vitro* dI4/dI5s recapitulate the same maturation trajectory as their endogenous counterparts.

### Functional analysis of dI4/dI5 subtypes reveal autism signatures in the spinal cord

We next used gene expression profiles within each cluster to infer the function of the *in vitro* dI4/5 subtypes. We projected the *in vitro* datasets onto the neural atlas to permit us to assign the stem-cell derived neurons into our previously annotated clusters (Fig. 1c, 6f). We then performed marker gene analysis on each of the day 43 *in vitro* dI4/dI5 subclusters followed by gene ontology, as previously performed on the *in vivo* dI4/dI5 subclusters (Fig 2e, f). Consistent with this analysis, the cross comparison of the resulting GO Biological Process terms among *in vitro*-derived dI4 and dI5 clusters, identified both shared and unique behavioral functions (Fig. 6j, k). Shared functions include pain perception, mechanosensory behavior, control of balance and regulation of neurotransmitter levels. Unique functions include subsets of dI4s (clusters 19, 20 and 29; Fig. 6f, j) that respond to cocaine, and a subset of dI5s that respond to temperature stimulus (cluster 13; Fig. 6f, k). Moreover, many functions, including pain and touch perception, the response to epinephrine and cocaine, and the regulation of balance, are shared between the *in vitro* and *in vivo* datasets. However, the specific cluster assignments for these functions generally differed between the two systems.

To further assess the molecular signatures within the clusters, the genes that made up the GO terms in the *in vivo* and in vitro clusters were subjected to a STRING analysis^75^, which infers function from predicted protein-protein interaction networks. We thereby identified the protein networks in both *in vivo* and day 43 *in vitro* clusters that encode pain perception (dI4s, Extended Fig. 7a, d, e, h, i; dI5s, Extended Fig. 9a, b, c, f), the response to cocaine (dI4s, Extended Fig. 7b, c, f), perception of mechanical stimulus (dI4s, Extended Figs. 7e and 8a, b, d, e; dI5s, Extended Fig. 9e) mechanosensory behavior (dI4s, Fig. 7l, n) and balance (dI4s, Fig. 7o, Extended Fig. 8c; dI5s, Fig. 7m). Consistent with the assessment that day 43 *in vitro* neurons are relatively immature, i.e. week 6 (Fig. 6h, i), the *in vitro* protein interaction networks are generally less complex than the *in vivo* networks. Nonethless, several *in vitro* networks share molecular signatures with the *in vivo* networks encoding the same function. For example, the *in vitro* and *in vivo* dI4 networks encoding pain perception in cluster 20 share a protein loop centered on NPY1R, which is thought to mediate neuropathic pain^76^ (Extended Fig. 7d, e). Similarly, *in vitro* dI4 touch circuit in cluster 4 (Extended Fig. 8b) appears to represent a simplified version of the *in vivo* dI4 touch circuit within the corresponding cluster (Extended Fig. 8a).

Moreover, we also found two highly correlated protein networks *in vitro* and *in vivo* clusters. *In vitro* cluster 4 encodes both mechanosensory behaviors (Fig. 6n) and balance (Fig, 6o), with molecular/functional signatures that closely parallel those found in *in vivo* cluster 9 (Fig. 6l) and cluster 49 (Fig. 6m), respectively. Remarkably, these signatures are enriched with genes with high SFARI scores^25^, which connate genes highly associated with autism spectrum disorder (ASD). These genes include NGLN2^77, 78^, NRXN1/2^77, 79^, SHANK1/3^80^, DLG3^81^, CNTNAP2^82^ which all have allele variants thought to predispose individuals to ASD. Given that individuals with ASD frequently report aberrant mechanosensation and balance, the presence of these transcriptional signatures in the *in vivo* atlas suggests that ASD-related molecular programs are present in the developing spinal cord, in addition to the brain. Notably, these signatures emerge as early as week 6 *in vitro*, supporting the possibility that spinal-cord-intrinsic mechanisms contribute to the manifestation of ASD phenotypes.

## Discussion

In this study, we establish multiple human NMP–based directed differentiation protocols to generate dIs from hESCs and benchmark their identities against a human spinal cord single-cell reference atlas. Integration of the *in vitro-*derived datasets with *in vivo* developmental trajectories reveals the NMP–derived human spinal neurons faithfully recapitulate endogenous human spinal cord identities and developmental timing. Moreover, NMP-derived dI4/dI5 subtypes have transcriptional programs that parallel their *in vivo* counterparts, consistent with their developing the appropriate sensory functions. Notably, we find networks that are enriched for high-confidence ASD risk genes, raising the possibility that spinal cord–intrinsic sensory circuits contribute to autism-associated sensory phenotypes. Finally, we show that distinct differentiation strategies collectively generate the full complement of dI populations, and that time and posteriorizing signals are key determinants of human spinal neuron diversification. Together, these studies provide an important resource for interrogating human spinal cord development and establish a conceptual framework for the rational design of cellular replacement strategies for sensory circuit regeneration following spinal cord injury.

### Role of time and GDF11 in posterior patterning

Temporal progression emerges as a key factor determining the posterior spinal cord identity *in vitro*. Previous studies showed that deterministic posterior HOX patterning can be achieved by extended NMP culture and GDF11 treatment^18^. We also find that prolonging NMP culture drives posteriorization. However, our studies also suggests that extended time in culture alters NMP identity in more complex ways, such as inducing the progressive enrichment of mesodermal gene signatures (Fig. 4). Thus, while posterior competence increases with time, prolonged NMP maintenance may simultaneously bias lineages, underscoring a tradeoff between axial maturation and progenitor plasticity.

Our unbiased bulk RNA-Seq analysis of NMPs cultured for different durations also did not reveal a strict co-linear activation of *HOX* genes, as observed previously^18, 83^. Rather, the 4d and 10d NMP cultures exhibited enrichment of broad, overlapping expression of multiple *HOX* genes, rather than the sequential activation of posterior *HOX* paralogs (Fig. 4). This result suggests that posteriorization in humans may require the combinatorial activity of multiple *HOX* genes rather than a single posterior HOX determinant. This finding further aligns with recent analyses of human fetal spinal cord development, which similarly report a breakdown of classical *HOX* co-linearity during human neurodevelopment^28^.

The role of GDF11 is more complicated and context dependent. In our study, GDF11 applied at during 10d NMP induction generally has minimal effects on gene expression (Fig. 4k) but may change the profile of *HOX* gene expression (Fig. 4o). This finding contrasts with an earlier study, which showed that treating 6d NMPs with GDF11 results in the robust induction of the most posterior *HOXA11/HOXA13* genes^18^*.* These two protocols also use different media, making it possible that differences in experimental conditions and developmental timing may explain this discrepancy. However, we observed posteriorizing and patterning effects when GDF11 was applied at the neural progenitor (dP) stage within EBs, rather suggesting a different timeframe of competence for GDF11. Thus, GDF11 appears to be most effective as a posteriorizing factor, when added after prior temporal patterning of NMPs.

Taken together, these findings suggest posterior identities in human stem cell–derived spinal neurons emerge through the interaction of developmental time with signaling environments, rather than from a single posteriorizing signal. In this model, GDF11 does not function as a binary posterior fate switch; rather, it acts as a modulatory signal that reinforces axial identity and patterning choices once progenitors have acquired sufficient posterior competence. This model is consistent with the prolonged timeline of human spinal cord development^84^ and the extended plasticity of human neural progenitors, possibly due to prolonged notch activity^85^ or non-coding RNAs, which could allow multiple signaling inputs to converge before specific dI fates are stabilized. Importantly, these observations provide mechanistic insights into how human spinal cord patterning can differ from the signaling hierarchies inferred from model organisms that have shorter gestation periods^86^.

### Developmental expansion and specialization of human dI4/dI5 populations

The human spinal cord reference atlas generated in this study offers an unprecedented view of interneuron diversity across developmental time and serves as a powerful platform for mechanistic discovery. One of the most striking features of the atlas is the dramatic expansion of dI4 and dI5 populations at later gestational stages (Fig. 2a and Extended data figure 2). Comparative analysis with a recently described mouse single cell atlas of the developing spinal cord^63^ suggests substantial overlap between human dI4/dI5 clusters and the late-born mouse dIL_A_/dIL_B_ subtypes.. In our analyses, the human dI4/dI5 subtypes have continuous lineage trajectories stemming from the progenitor population and share molecular identities with canonical dI4/dI5 markers. We thus propose that late-born dIL_A_/dIL_B_ are equivalent to late-born dI4/dI5s.

dI4/dI5s are among the most clinically relevant spinal interneuron populations, playing central roles in processing pain, touch, and itch. The *in vivo* reference atlas reveals biologically relevant gene expression programs that suggest modality-specific sensory functions within these populations. For example, the dI5–3 population is enriched for mechanosensory-associated genes such as PIEZO2, while dI4–3 population expresses NPY, an inhibitory neuropeptide implicated in pain processing. We also identify many transcriptionally distinct populations that do not overlap with the mouse populations, suggesting there are dI4/dI5 subclusters with human specific sensory functions. Identifying these functions requires further investigation, however species-specific differences in somatosensory modalities have been observed, such as the ability of crocodilians species to sense small, local pressure changes in water to thereby detect their prey^87^. Moreover, the mechanistic basis for somatosensory differences between individuals remains an intriguing and understudied area. Subtle biases within dI4/dI5 populations could explain pain perception differences between individuals, or the development of the proprioceptive acuity observed in elite athletes.

Together, these analyses reveal a previously underappreciated diversity in human somatosensory circuit organization and underscores the value of temporally resolved human atlases for uncovering clinically relevant cell types critical for neural repair.

### Role of spinal cord circuitry in mediating ASD phenotypes

One of the most unexpected findings from the reference atlas is the identification of gene signatures in dI4/dI5 neuronal populations related to ASD risk pathways (Fig. 6–o, Extended data figures 7–9). Both *in vitro* and *in vivo* dI4/dI5 clusters contained biological processes related to pain perception, mechanosensory behavior, balance, and neuromodulatory responses. Consistent with the observation that the *in vitro* neurons resemble an early developmental state (GW6–8*), in vitro* networks were less complex than *in vivo* networks, but nonetheless captured core molecular signatures of corresponding *in vivo* circuits. Notably, a subset of *in vitro* and *in vivo* dI4/dI5 clusters associated with mechanosensation and balance were enriched for highly associated ASD-risk genes, including NRXN1^88^, SHANK1/3^89^, DLG4^90^, CNTNAP2^91^, and NLGN2^92^. Given the prevalence of differences in touch and balance processing in autistic individuals, the presence of these transcriptional and network-level signatures in the developing human spinal cord suggests that ASD-associated molecular mechanisms may not be confined to the brain. The identification of these signatures in *in vitro*-derived interneurons supports the idea that ASD-linked changes arise during embryonic development ^93^ and that developmental processes intrinsic to the spinal cord contribute to ASD-linked sensory phenotypes, alongside brain-specific mechanisms.

### Implications for spinal cord injury and regenerative therapies

The insights gained from this study have direct relevance for SCI research and regenerative medicine. Sensory dysfunction after SCI arises both from neuronal loss and damage to the dI circuits that integrate and relay somatosensory information. The human spinal cord reference atlas shows that dI4/dI5 populations continue to expand during development, ultimately outnumbering the other dI classes such that they constitute the major cellular components of human dorsal sensory circuits. Notably, our stem cell–derived cultures recapitulate this bias, generating much larger population of dI4/dI5s but more limited numbers the other classes of dIs. This concordance in neural ratios provides an important benchmark for regenerative strategies, suggesting that these differentiation protocols more faithfully reproduce the cellular composition of human spinal cord and may thus be well suited for reconstructing dorsal spinal pathways lost after injury. Together with the observation that our stem cell-derived human dIs are transcriptionally indistinguishable from their endogenous counterparts (Fig. 6d, e), these studies provide a rational strategy for directing donor iPSC-derived cells towards dI subtypes that are matched for axial identity, and developmental stage. This framework marks a critical step toward a precision medicine approach for treating spinal cord injuries.

### Limitations and future directions

Despite these advances, several questions remain. Some dI populations, most notably the dI2s, remain hard to detect in the *in vitro* protocols, outside of scRNASeq approaches. This issue raises the possibility that there are technical limitations identifying these populations, or that these neuronal identities exist in a transient developmental window which evade detection. In addition, the *HOX* gene expression patterns in the *in vitro*-derived NMPs and dIs *in vitro* do not follow a simple anterior–posterior hierarchy. This observation suggests that axial identity may be governed by combinatorial or context-dependent mechanisms during human neural tube development and needs further investigation. Finally, to advance these studies towards a cellular therapy, further studies are needed to assess maturation-promoting strategies within the protocols^94^ and to determine whether the *in vitro*-derived dIs can restore function when integrated into an SCI model.

## Methods

### Construction of the human atlas

#### Integration of single-cell datasets from different origins

Data from six publications^28, 50–54^ were loaded into Seurat v5^57^ in R. For each dataset, all samples were combined into one object, and each sample was split into a separate layer. Quality control was performed automatically by removing cells for each sample that fell outside of three median absolute deviations (both sides for nCount_RNA and nFeature_RNA, and higher for percent.mt). The Anderson dataset did not include mitochondrial genes and thus was only filtered on the two other metrics. After QC, all datasets were combined into one large Seurat object and the data was normalized and variable features were calculated. The object was then converted to anndata and integration was performed using SCVI^58^ on the counts layer using only the variable features (n_layers = as.integer(2), n_latent = as.integer(30), gene_likelihood = “nb”). The latent representation was then brought back into the full Seurat object and clusters and UMAP were calculated. Cluster identities were assigned using sctype^59^ and a custom list of marker genes collated from various publications.

#### Reclustering of neuronal clusters

To analyze the neuronal population, clusters identified as neurons, peripheral neurons, sensory neuron progenitors, and progenitors-neurons were isolated from the atlas and reintegrated using newly calculated variable features and new scVI parameters as recommended by scArches^74^ (use_layer_norm = “both”, use_batch_norm = “none”, encode_covariates = T, n_layers = as.integer(2), dropout_rate = as.numeric(0.2)). After dimensional reduction, a few undesired populations were still left, so clusters that were identified either as peripheral populations, or unidentified neuronal populations (mostly identified by a lack of distinct marker genes) were removed and the dataset was reintegrated.

#### dI cell type extraction based on the marker gene expression

To identify the various populations of cells within the neuronal atlas, AUCell^62^ was used with a custom list of marker genes to create a module score for each cell, for each possible cell type. Cutoffs were manually assigned for scores for each cell type, and every cell was assigned 0, 1, or multiple identities. Cells with multiple identities were then rectified using a kNN classifier (k=10) to change their identity to match those of surrounding cells. Then the same was done with cells assigned no identity to extrapolate to nearby populations not expressing high levels of any module.

#### dI4/dI5 subcluster analysis

Differential gene expression analysis across clusters was performed using the FindAllMarkers function from the Seurat R toolkit. The results were filtered, to only include clusters identified as containing large portions of either dI4 (2, 4, 5, 7, 9, 11, 12, 14, 18, 19, 20, 23, 26, 27, 29, 36, 37, 43, 44, 48, 52, 65) or dI5 (0, 10, 13, 15, 16, 17, 21, 30, 49, 54, 61). Of note is that due to the AUCell classification process, clusters are not made up exclusively of one cell type, but each cluster identified here has a large portion of cells identified as either dI4/5. Gene Ontology (GO) enrichment analysis was performed on significantly upregulated genes in each cluster (avg_log2FC > 0.25) using the enrichGO function from the clusterProfiler R package. The ontology category was restricted to Biological Process (BP), multiple testing correction was applied using the Benjamini-Hochberg method, and terms were considered significant with an adjusted p-value of 0.05 and q-value of 0.2. GO categories for dot plot visualization were selected based on their association with various sensory modalities and overlap, or lack thereof, amongst clusters. The STRING (Search Tool for the Retrieval of Interacting Genes/Proteins) resource was utilized to explore known and predicted protein-protein interaction networks from genes that were making up GO categories of interest.

### Single-cell RNA sequencing and analysis of in vitro cultures

#### Preparation of single cell suspension

Single cell suspension from EBs is generated via papain dissociation method using the Worthington papain dissociation kit (refer to manual). Briefly, EBs are first collected in 15mL centrifuge tubes and washed twice with DPBS. The papain solution is prepared by mixing papain with DNAse (supplied in the kit), and the solution is allowed to activate for 10 minutes at 37°C before adding to the EBs followed by triturating 10 times with a p1000 pipette. EBs are then preferably placed on an orbital shaker at 37°C and 5% CO_2_ for 1 hour and 15 minutes and triturated 10 times every 5 minutes or until no visible EB clumps remain. Single-cell suspension of EBs are then centrifuged at 300 × g for 7 minutes. The cell pellet is resuspended in 2mLs of Ovomucoid protease inhibitor solution to stop the papain digestion and passed through a 40μm cell strainer to remove undigested organoid debris. Single cells are then centrifuged once more at 300 × g for 7 minutes and the cell pellet is then resuspended in 1mL of 0.04% BSA in DPBS for GEM preparation using 10X genomics platform (the concentration of BSA can be increased to reduce cell stickiness/chance of doublets).

#### Library preparation, and sequencing

~ 10,000 live cells/condition were used to generate single-cell cDNA libraries using protocol described by the 10X Genomics 3’GEX library preparation kit. Cells were first partitioned into GEMs (gel-beads-in-emulsion) containing using the 10x Genomics Chromium controller. Further, 3’ mRNA-seq gene libraries were prepared using the Chromium Single Cell 3′ Library & Gel Bead Kit v2 (10x Genomics), according to the manufacturer’s instructions. Library quality control was assessed using Agilent and Qubit assays. If quality control is within acceptable conditions (e.g., library concentration of 20–40ng/ul), libraries were sequenced on the Illumina NovaSeq X Plus 10B. For 3’GEX libraries targeting 10,000 cells 1,800 million reads/sample were obtained.

#### Integration of single-cell in vitro datasets

Single-cell libraries were processed with the 10x Genomics Cell Ranger Count pipeline (v9.0.1) and loaded into Seurat v5 in R. Separate Seurat objects were created for the RA/BMP series without GDF11 and the RA ± GDF11 series. For each object, Quality control was performed for each sample by removing cells that fell outside of three median absolute deviations for total UMI counts (nCount_RNA) and number of detected genes (nFeature_RNA), and above three median absolute deviations for percent mitochondrial reads (percent.mt). After QC, data were normalized and variable features calculated independently. The two objects were then merged into a single Seurat object.

For nonlinear batch integration, the dataset was subset to variable features and converted to an AnnData object. Integration was performed with SCVI, using a model trained on raw counts with default settings (batch_key = “sample”), to obtain a low-dimensional latent embedding. The latent representation was imported back into Seurat as a dimensional reduction, and was used to compute nearest-neighbor graphs, clusters, and UMAP embeddings. To provide an independent annotation of broad neuronal versus non-neuronal populations in the in vitro cultures, we applied sctype with a custom list of marker genes collated from various publications. For downstream atlas-based analyses, the dataset was restricted to cells classified by sctype as neuron, peripheral neuron, sensory neuron progenitor, or progenitor–neuron.

#### Projection of in vitro cells onto the human atlas

Projection of the in vitro cells onto the human spinal cord atlas and its neuronal subset was performed using pretrained SCVI reference models built on the whole in vivo atlas and the neuronal atlas respectively. The in vitro Seurat object was subset so that its gene set matched the genes present in the reference AnnData object. After conversion of the object to AnnData, reference mapping followed the scArches workflow (SCVI.prepare_query_anndata followed by SCVI.load_query_data), and the query model was fine-tuned for 200 epochs to align the in vitro dataset to the reference latent space. The resulting query-aligned latent representation was imported into Seurat and projected into the appropriate reference UMAP space using the saved UMAP models stored in the atlas objects.

To transfer annotations from the *in vivo* neuronal atlas to the in vitro cells, we trained supervised machine-learning models using atlas SCVI embeddings as predictors. K-nearest neighbors (k = 10) was used for dI cell-type classification (based on AUCell-derived identities; see dI cell type extraction based on the marker gene expression) and prediction of developmental week, while random forest (ranger) was used for cluster identity transfer. Cluster-specific markers for cells assigned to in vivo dI4 or dI5 enriched clusters were identified using FindAllMarkers. These gene sets were used to assess if in vitro cells recapitulate in vivo dI4/dI5 subtype transcriptional programs (see dI4/dI5 subcluster analysis).

### Bulk-RNA sequencing and data processing

#### RNA preparation

NMP cultures subjected to bulk-RNA sequencing were first washed with 1X DPBS to remove the media. Cells were then lysed by directly adding 600μL of RLT buffer in the culture wells, and the lysate is then collected and flash frozen with liquid nitrogen to be stored at −80°C. The total RNA was then purified using Qiagen’s RNeasy kit, and eluted in 50μL of RNAse-free water. Using a microvolume spectrophotometer, RNA concentrations were determined and diluted to 1μg/μL. RNA samples were further subjected to quality control assessment using Agilent Technologies 2100 Bioanalyzer and only samples with a RIN score >8.0 were used for library construction using Universal plus mRNA sequencing kit (NuGEN). Libraries were then sequenced on an Illumina Novaseq X Plus to generate ~30 million reads per sample.

#### Processing and differential expression analysis

Reads were obtained as raw FASTQ files, assessed for quality with FASTQC, and aligned to the human reference genome (GRCh38, GENCODE v29 annotation) using STAR. Gene-level counts were obtained with featureCounts (exon-level, paired-end read counting). Differential gene expression was tested using DESeq2. For each reference level of interest (hESC, D2NMP, D4NMP, D10NMP), a re-leveled model was built and processed separately using the design formula ~ condition, to obtain pairwise contrasts across all conditions. Global transcriptional relationships among samples were assessed using VST-normalized counts from the hESC-referenced model for PCA analysis, sample-to-sample Euclidean distance calculations, and heatmaps.

Shrinkage of log_2_ fold-changes for volcano plots and Gene Ontology (GO) enrichment analyses was performed on contrasts using apeglm. For each contrast, significantly upregulated and downregulated gene sets (padj < 0.05, |log_2_ fold change| > 1) were analyzed separately with clusterProfiler (enrichGO) using the background of all detected genes in the experiment. The ontology category was restricted to Biological Process (BP), p-values were adjusted using Benjamini–Hochberg, and terms with adjusted p < 0.05 (q < 0.2) were considered significant.

### Cell culture

#### ESC maintenance and culture

H9 ESCs were maintained in mTeSR plus media and passaged at least twice post-thaw onto Matrigel coated plates before NMP induction. ESCs were passaged when they reached 70% confluency using ReLeSR and were plated at a 1:4 dilution ratio. The newly passaged hESC cultures reached 70% confluency within 4 days.

#### NMP induction

To induce 2-, 4-, and 10-day NMPs, hESCs were cultured in a N2/B27-based SAND media without vitamin A (IMDM, N-2 1%, B-27 without vitamin A 2%, GlutaMAX 1x, NEAA 1x) under adherent culture conditions. Briefly, NMP induction was initiated by first pulsing the hESCs with 10ng/mL of bFGF for one day followed by the combination of bFGF + Wnt agonist, CHIR 99021. For example, to derive 2-, 4-, or 10-day NMPs, the differentiating hESCs received one day of 10ng/mL bFGF, followed by bFGF + 3μM CHIR 99021 for one day (2-day NMPs), three days (4-day NMPs), or nine days (10-day NMPs) respectively.

#### Embryoid body (EB) differentiation

Upon the completion of NMP induction in adherent cultures, cell sheets containing NMPs were cut with an EZ passage tool and transferred to the ultra-low attachment plates which allowed NMP clumps to self-aggregate into EBs. EBs were cultured with SAND media containing vitamin A (SAND + B27 with vitA) and further supplemented with 1μM RA for two days to induce neural patterning. From the 2nd day until the 14th day, EBs were treated with various patterning factors to induce relevant cell types. For example, to induce dI4–6 populations, EBs were treated with 1μM RA ± GDF11 up to 14 days in suspension cultures. For inducing dI1–3 cell types, EBs were bathed in SAND media supplemented with 1μM RA + 10ng/mL BMP4, and ± GDF11 (50/100ng/mL). On the 14th day EBs were switched into maturation media until day 20, day 43, or day 130 and sampled for quality control checkpoints during maturation. Media changes were performed by collecting EBs in 15mL conical tubes and allowing them to sink to the bottom of the tube in a pellet. Exhausted media was aspirated and replaced with fresh media corresponding to condition. EBs were then transferred back to the plates using glass borosilicate pipettes which prevented EB loss due to sticking to the walls of the plastic pipettes.

#### Immunocytochemistry

NMPs:Immunocytochemistry (IHC) was performed on NMPs to determine the expression of NMP markers such as SOX2 and Brachyury (T). Briefly, at the end of NMP culture durations, the sheet of NMP cells was cut into pieces by EZ Passage tool and transferred onto Matrigel coated 8-well Ibidi slide chambers. In Ibidi slides, NMPs were allowed to grow for another day in their respective condition media and were processed for IHC the following day. NMPs were washed once with 1X DPBS and then fixed on ice with 4% PFA for 8–10 minutes. PFA solution was removed by three 5-minute washes with 1X DPBS, followed by incubation in the antibody blocking solution (1X DPBS, 1% HIHS, 0.1% Triton X-100, and 0.05% sodium azide) for 1 hour at 2–8°C. Primary antibodies were diluted in antibody blocking solution and added to the NMPs for the overnight incubation at 2–8°C. The following dilution was used: SOX2 (mouse; Santa Cruz, sc-365823; 1:500), BRACHYURY (goat; R&D systems, AF2085; 1:500), and CDX2 (rabbit, Cell Signaling Technology, D11D10; 1:500). NMPs were washed twice with 1X DPBS with 0.1% Triton X-100 (PBT) for 5 minutes before secondary antibody incubation. Corresponding species-specific secondary antibodies (Jackson ImmunoResearch Laboratories) were diluted 1:400 in 1X PBT and allowed to incubate in the dark at room temperature for 1 hour followed by counterstaining with nuclei stain DAPI (1:500 dilution in 1X PBT) for 15 minutes and replaced with 1X DPBS for imaging.

##### Embryoid bodies (EBs):

EBs were first collected by the sinking method, and the media was removed by two 5-minute washes with 1X DPBS, followed by fixing in 4% PFA on ice for 10 minutes on rocker. EBs were then washed three times for 5 minutes before allowing them to sink in 30% sucrose in 1X PB solution for at least 1 hour or until all the EBs had sunk to the bottom of the tube, indicating adequate equilibrium with sucrose had been reached. EBs were then embedded in OCT on dry ice blocks, and then sectioned at 14μm thick sections using Leica Cryostat onto positively charged microscope slides. Slides with EB sections were then washed with 1X DPBS, blocked with the antibody blocking solution for 1 hour at room temperature before receiving primary antibodies. The following antibodies were used: LHX2 (mouse; DSHB, PCRP-LHX2–1C11; 1:50), FOXD3 (guinea pig, a gift from Thomas Mueller, Germany, 1:10,000), ISL1 (goat; R&D systems, AF1837; 1:500), PAX2 (rabbit; Invitrogen, 71–6000; 1:500), LMX1B (guinea pig; Thomas Muller Lab), LHX1/5 (mouse; DSHB, 4F2; 1:50) for 12–14 hours (overnight) at 2–8°C in a humidified chamber. Slides were washed twice with PBT before receiving species-specific secondary antibodies (Jackson ImmunoResearch) 1:400 in the dark for 1 hour at 20°C. Slides were then washed once with 1X PBT for 5 minutes and incubated with DAPI for 15 minutes to counterstain the nuclei. Slides were then mounted with Prolong Diamond antifade medium and imaged using a Zeiss LSM800 inverted confocal microscope.

#### Quantitative (q) RT-PCR analysis

Samples of interest were lysed with ~300–500μL RLT buffer and total RNA was purified using Qiagen RNeasy Kit. Total RNA was quantified on a microvolume spectrophotometer, and 500ng/μL RNA was used to generate cDNAs using Superscript IV First Strand Synthesis kit. The gene-specific primers (forward and reverse) were used at 0.5μM final concentration in a 10μL reaction with SYBR Green and qRT-PCR was performed on Roche 480 Light-cycler (Roche). The relative fold expression was determined using the ΔΔCT method to compare the expression of the target gene against the expression of a housekeeping gene glyceraldehyde 3-phosphate dehydrogenase (Gapdh).

## Extended Data

**Extended Data Figure 3:**
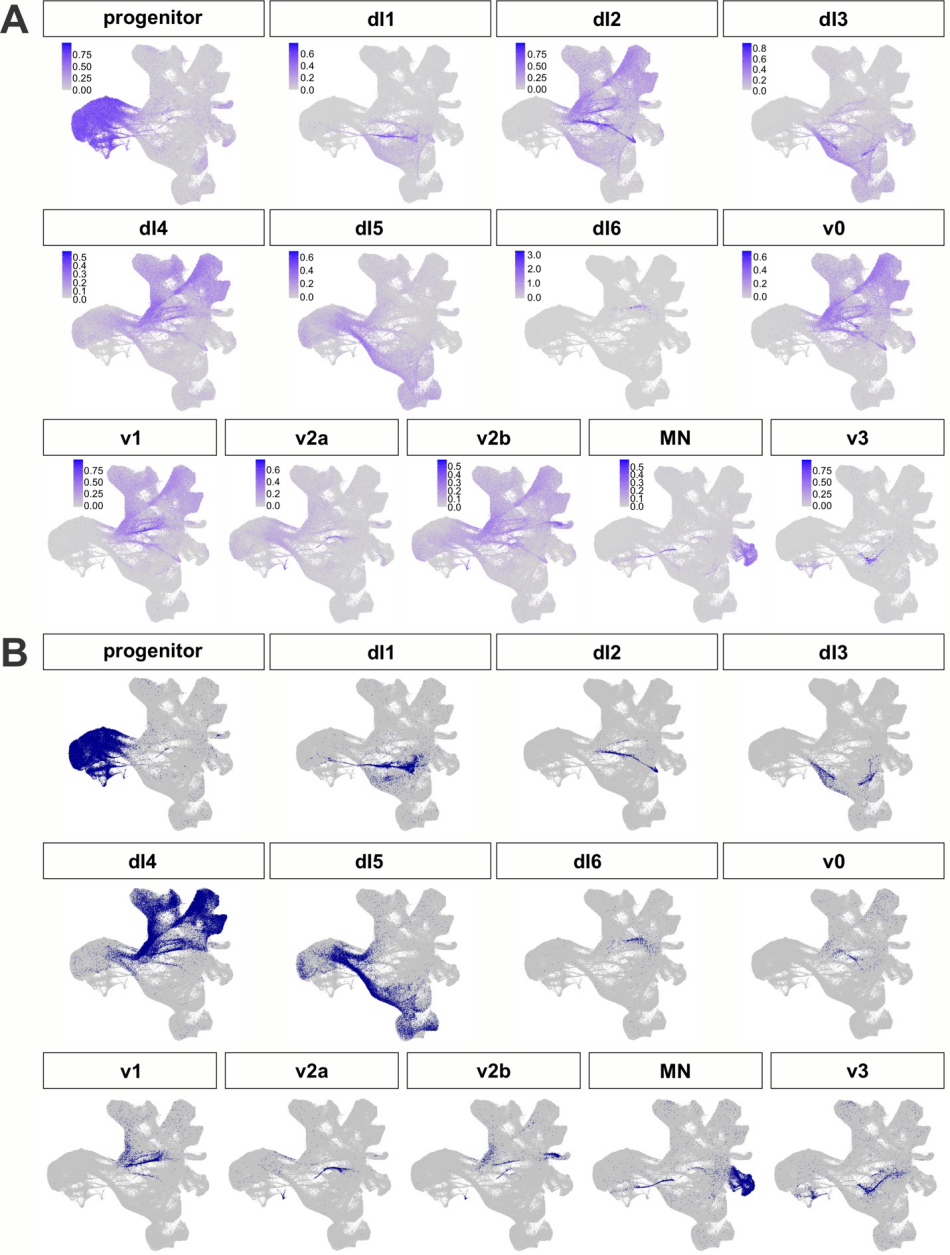
Assignment of spinal trajectories within the *in vivo* atlas (A) AUCell was used with a custom marker gene list to plot module scores for each neuronal lineage in the atlas. For dI6, expression of DMRT3 is shown since AUCell did not provide sufficiently specific scores. (B) Cutoffs were manually assigned for each cell type to determine which cells could be classified as each cell type. Cells with multiple identities were then rectified with kNN analysis, and then identities were extrapolated to cells with no identity using kNN analysis as well.

**Extended Data Figure 4:**
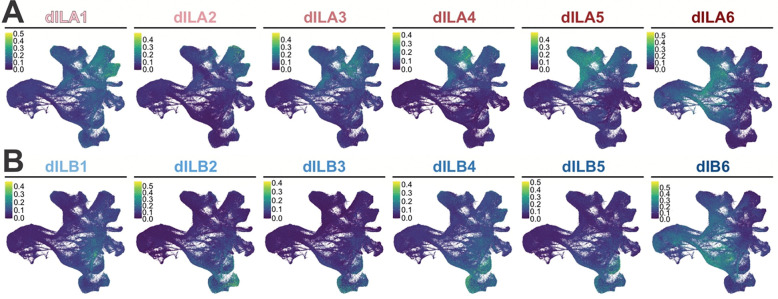
Assignment of mouse dI4/dI5 identities on human neural atlas (A-B) Gene lists from Roome et al^63^ of each dIL subtype were used in AUCell to calculate module scores for each of the potential dIL subtypes. This was then used to map their general location onto our atlas.

**Extended Data Figure 5:**
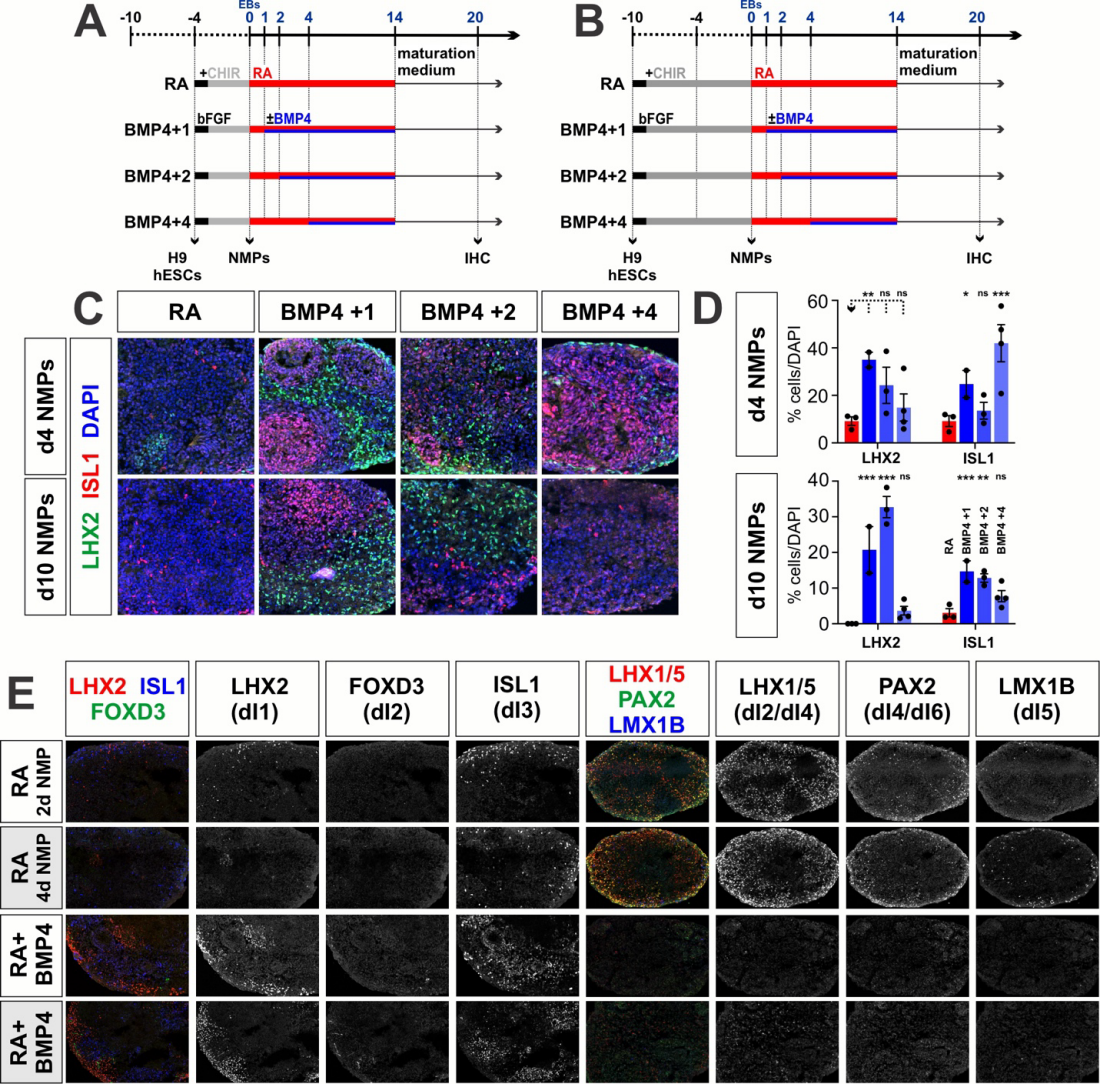
Assessing the timing of BMP4 addition in d4 and d10 NMP protocols. (A, B) EBs were generated at day 0 after either d4 (A) or d10 (B) NMP formation and then treated with RA for 10 days (control). In the experimental samples, BMPs were added at day 1 (BMP4+1), day 2 (BMP4+2) or day 4 (BMP4+4) after EB formation, until day 10. Samples were further differentiated for 6 days and then processed for immunohistochemistry (IHC). (C) EBs were labeled with antibodies against LHX2 (dI1, green), FOXD3 (dI2, white), ISL1 (dI3, red) and DAPI (all nuclei, blue). (D, E) Quantification of EBs formed from either d4 (D) or d10 (E) NMPs, suggests that dI1s form robustly from the BMP4+1 condition for both d4 and d10 NMPs. In contrast, dI3s form most robustly from BMP4+4 condition for d4 NMPs and the BMP4+1 condition for d10 NMPs. Probability of similarity between control and experimental groups: * p<0.05, ** p<0.005, *** p<0.0005; two-way ANOVA.

**Extended Data Figure 6:**
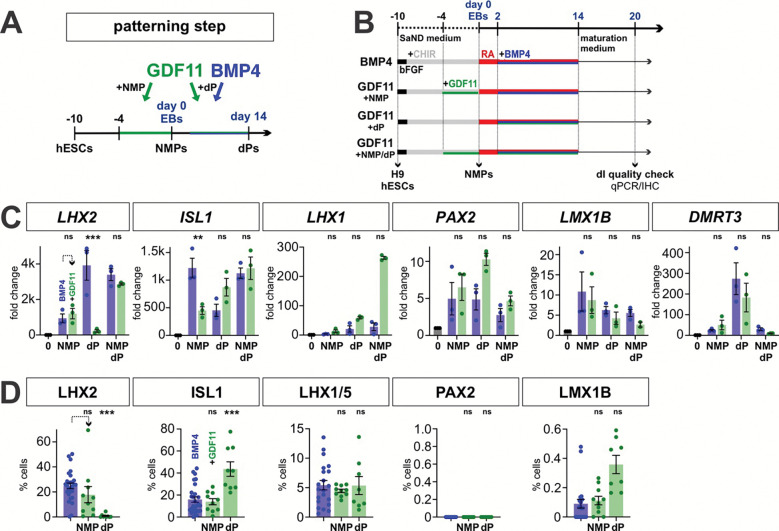
Effect of GDF11 on dI1 identity in the RA+BMP4 protocol (A, B) Overview of the experimental timeline/workflow for the RA±BMP4±GDF11 NMP protocols. The effect of GDF11 treatment was assessed on NMP patterning, dP patterning and both NMP and dP patterning (A). (C, D) qPCR (D) and IHC (D) analyses of day 20 EBs, suggest that the addition of GDF11 together with BMP4 at the dP stage significantly decreases LHX2^+^ dI1 identity, but increases ISL1^+^ dI3 identities. There are no other significant effects. Probability of similarity between control and experimental groups: **p<0.005 *** p<0.0005.

**Extended Data Figure 7:**
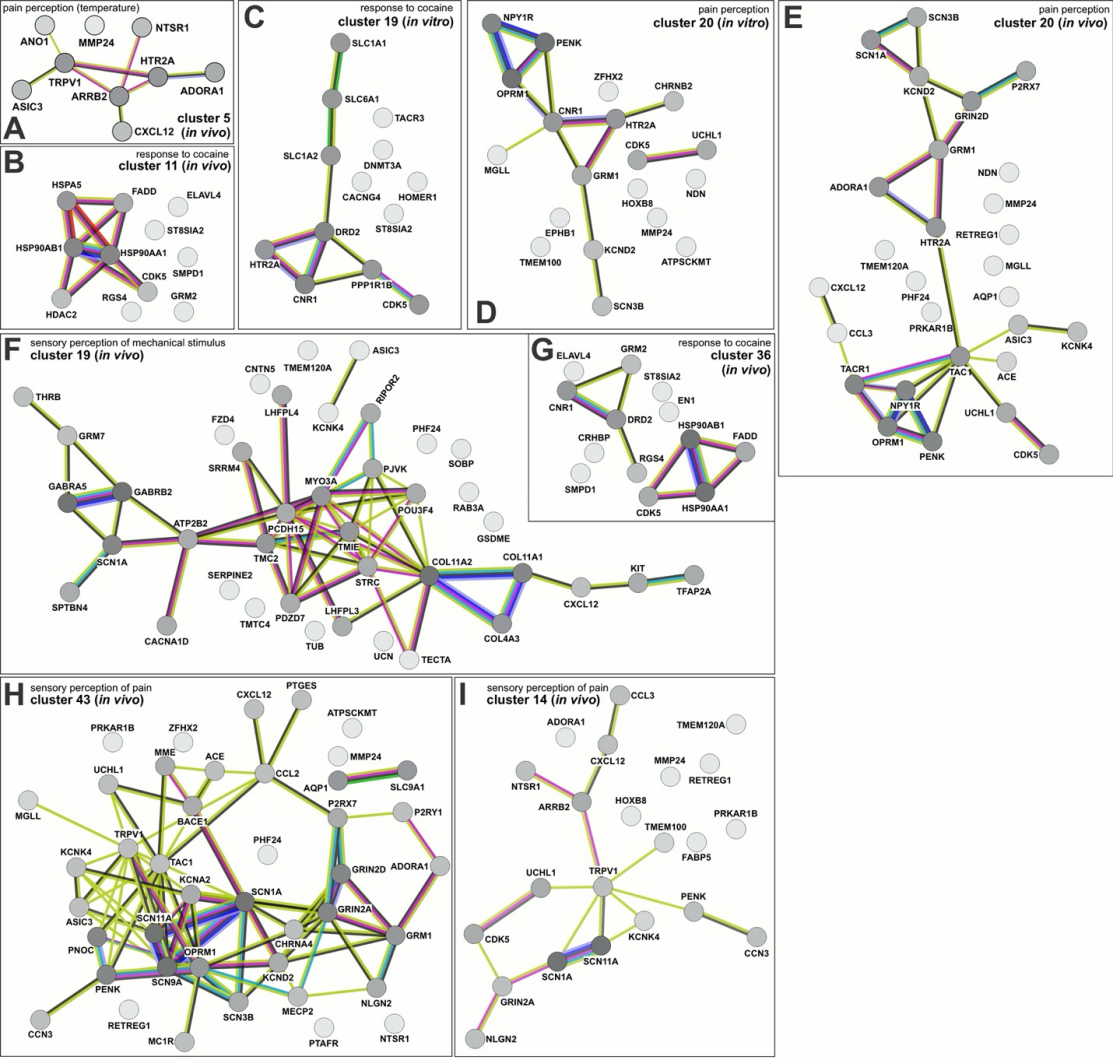
Pain perception, cocaine response, and mechanosensory networks are found in *in vivo* and *in vitro* dI4 clusters The STRING (search tool for the retrieval of interacting genes/proteins)^75^ was used to identify sensory modality networks from genes upregulated in both *in vivo* and *in vitro* dI cell populations. Nodes represent proteins, the lines (or edges) between the nodes represent protein-protein associations. Numerous edges between nodes implies greater evidence (experimentally determined or predicted) for protein-protein associations. STRING further documents evidence of protein associations through the edge color as follows: Known interactions: experimentally determined (purple) or from curated databases (teal). Predicted interactions: gene neighborhood (green), gene fusions (red), gene co-occurrence (blue). Others: text mining (yellow), co-expression (black), protein homology (periwinkle). (A, D, E, H, I) Networks associated with pain perception (A) *In-vivo* cluster 5 cells make up the sole network linking temperature with pain, centering around NTSR1, TRPV1, ARRB2 AND HTR2A. Pain circuits regulated by PENK, OPRM1, NPY1R, HTR2A and GRM1 are found in both *in vitro* cluster 20 (D) and *in vivo* cluster 20 (E). The *in vivo* cluster 43 (H) circuit has similarity with *in vivo* cluster 14 (I), sharing the SCN1A, SCN11A, and TRPV1 nodes. (B, C, G) Protein networks involved in response to cocaine. Both *in vitro* cluster 19 (C) and *in vivo* cluster 36 (G) circuits center around DRD2 and CNR1, further evidence supporting our *in vitro* generated dI4 cells may be functionally similar to their *in vivo* counterparts. (F) Sensory perception of mechanical stimulus protein network stemming from upregulated genes found in *in vivo* cluster 19

**Extended data figure 8:**
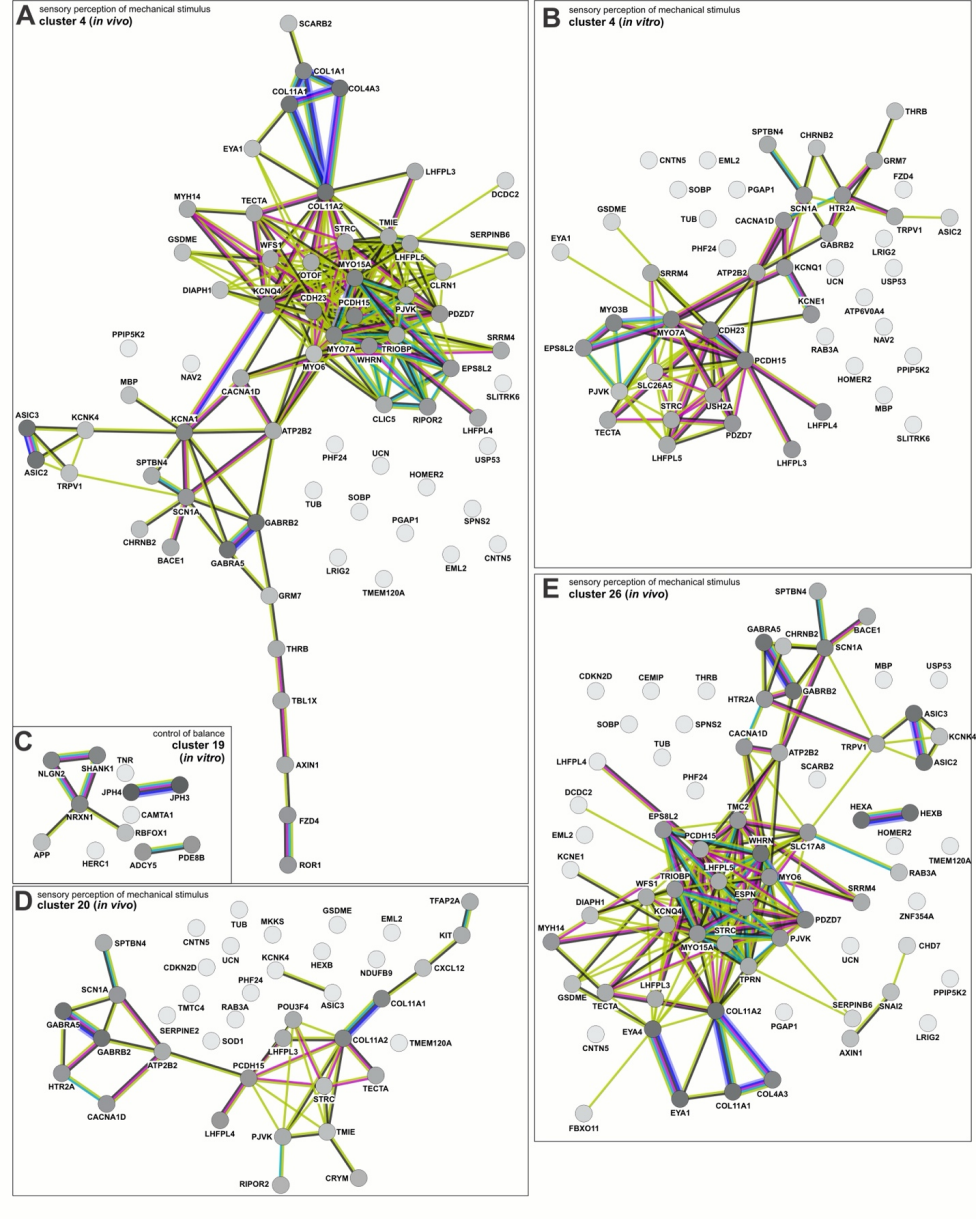
Mechanosensation and balance networks are found among *in vivo* and *in vitro* dI4 clusters (A, B, D, E) Numerous *in vitro* and *in vivo* clusters contain protein interaction networks involved in mechanosensation. The *in vitro* cluster 4 (B) circuit includes MYO7A, PCDH15, MYO3B and EPS8L2, key nodes also found across *in vivo* clusters 4 (A), 20 (D) and 26 (E). (C) A protein network associated with balance is found in *in vitro* cluster 19. Notably, this includes SHANK1, NLGN2, and NRXN1, genes with allele variants associated with autism spectrum disorder.

**Extended Data Figure 9:**
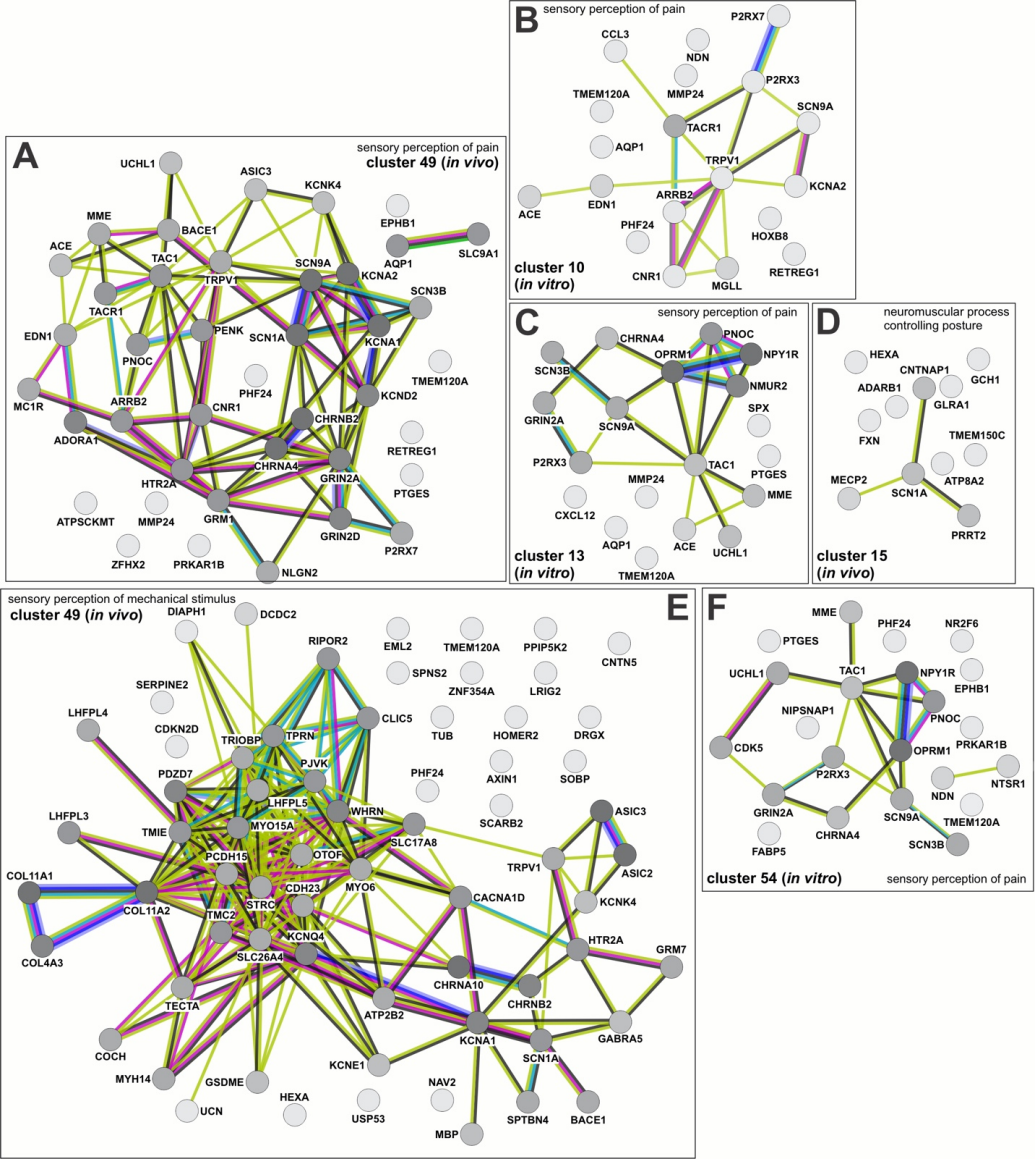
Pain perception, posture regulation, and mechanosensory networks are found among *in vivo* and *in vitro* dI5 clusters (A-C, F) Networks identified associated with pain perception. The cluster 49 *in vivo* circuit includes strong associations between SCN9A, SCN1A, KCNA1, and SCN3B. Part of this module is recapitulated in *in vitro* cluster 10 (B), with the presence of SCN9A and KCNA2 as well as *in vitro* cluster 54 (F) with the presence of SCN9A and SCN3B. *In vitro* cluster 13 (C) interestingly includes NMUR2, a protein crucial in sensing mechanical itch, which is not present in other identified networks. (D) Unique to the *in vivo* dataset is a neuromuscular posture regulating network comprised of CNTNAP1, SCN1A, PRRT2 and MECP2. (E) A large *in vivo* network associated with mechanosensation is found in cluster 49 and includes similar nodes such as PCDH15 described in the clusters in Extended data figure 8 relating to mechanosensation.

## Supplementary Material

Supplement 1
